# Changes and correlates of screen time in adults and children during the COVID-19 pandemic: A systematic review and meta-analysis

**DOI:** 10.1016/j.eclinm.2022.101452

**Published:** 2022-05-21

**Authors:** Mike Trott, Robin Driscoll, Enrico Irlado, Shahina Pardhan

**Affiliations:** Vision and Eye Research Institute (VERI), Anglia Ruskin University, Young Street, Cambridge CB1 2LZ, UK

**Keywords:** Covid-19, Screentime, Children, Adults, Review

## Abstract

**Background:**

Screen time has increased as a result of the COVID-19 pandemic, and several correlates have been associated with these increases. These changes, however, have not been aggregated. It was the aim of this review to (a) aggregate changes in screen time in adults and children, and (b) report on variables in relation to screen time during the COVID-19 pandemic.

**Methods:**

A systematic review of major databases was undertaken for studies published from inception to 06/12/2021, using a pre-published protocol (PROSPERO ID: CRD42021261422). Studies reporting (a) screen time pre-versus-during the pandemic, (b) screen time percentage change, or (c) correlates of screen time during the pandemic were included. A random effects meta-analysis was undertaken with subgroup analysis by age group and type of screen time.

**Findings:**

After review, 89 studies (*n* *=* 204,734; median age=20·6; median female=53·3%) were included. The majority of studies were cross-sectional. With regards to total screen time, primary aged children (6–10 years) reported largest increases (1·4 hrs/day; 95%CI 1·1–1·7), followed by adults (>18 years; 1·0 hrs/day; 95%CI 0·7–1·2), adolescents (11–17 years; 0·9 hrs/day; 95%CI 0·3–1·5), and young children (0–5 years; 0·6 hrs/day 95%CI 0·3–0·9 hrs/day). For leisure screen time (non-work/non-academic), primary aged children reported largest increases (1·0 hrs/day 95%CI 0·8–1·3), followed by adults (0·7hr/day 95%CI 0·3–1·2), young children (0·6 hrs/day; 95%CI 0·4–0·8), with adolescents reporting the lowest increase (0·5 hrs/day 95%CI 0·3–0·7). Several correlates were associated with reported increases in screen time, including adverse dietary behaviours, sleep, mental health, parental health, and eye health.

**Interpretation:**

Pooled evidence suggest that primary aged children reported the highest increase in both total and leisure screen time during COVID-19. It is recommended that screen time should be reduced in favour of non-sedentary activities. This study has the potential to inform public health policy and future guidance regarding screen time, and to inform future research in this area.

**Funding:**

No funding was received for this study.

**Research in context**

**Evidence before this study**

Sedentary behaviour has increased because of the COVID-19 pandemic. It was the aim of this review to pool and report on increases in screen time during the COVID-19 pandemic, and review associations between screen time and correlates. Pubmed, Embase, Scopus, PSYCInfo, ERIC, Child development and adolescent studies, Web of Science and Opengrey were searched using terms relating to “screen time”, “digital screen time”, “sedentary behaviour”, “sitting time” and COVID-19 from inception to 6/12/2021.

**Added value of this study**

This review found that all age groups increased their total screentime. Primary aged children (6–10 years) reported largest increases, followed by adults (>18 years), adolescents (11–17years), and young children 0–5 years). Leisure screen time also increased in all aged groups, with primary aged children reporting the largest increases, followed by adults, young children, and adolescents. Several correlates were associated with reported increases in screen time, including adverse dietary behaviours, sleep, mental health, and eye health.

**Implications of all the available evidence**

This review provides evidence that screen time should be reduced wherever possible to negate potential adverse outcomes, and, instead, non-sedentary activities should be promoted.

Alt-text: Unlabelled box

## Introduction

In March 2020, the World Health Organization (WHO) declared the COVID-19 outbreak a global pandemic, and as of 19th January 2022, over 325,000,000 confirmed cases have been diagnosed in more than 130 countries and areas, resulting in approximately 5500,000 deaths.^1^ Since the beginning of 2020, more than 100 countries have enforced some kind of social distancing measures to reduce the rate of COVID-19 transmission, commonly called ‘lockdown’.^2^ The severity of lockdown has varied from country to country, even region to region, with some countries limiting the distance people could travel from their homes, and some banning any unnecessary outdoor activity.^2^ These lockdowns have undoubtedly impacted the way in which people work, travel, and spend recreational time. For example, a systematic review reported in the initial phases (in the year 2020) of the pandemic, the majority of adults and children had increased time in sedentary behaviours, defined as any waking behaviour with an energy expenditure of ≤ 1·5 Metabolic Equivalents (METs) whilst in a sitting or reclining posture,^3^ with concurrent decreases in physical activity,^4^ although the study did not stratify the type of sedentary behaviour. One type of sedentary activity that has increased substantially during the pandemic is screen time – adults who are now working from home are increasingly using online platforms for work meetings, and children in a number of countries have been taking part in their educational classes online,^5^ with an estimated 1.37 billion children being at home in March 2020.^6^ To date, however, these changes in screen time have not been aggregated, which is likely due to a paucity of homogeneous data. On the other hand, several correlates have already been associated with increases in screen time in adults and children independently, including unfavourable dietary choices (such as positive associations between screen time and increases in alcohol and sweetened foods consumption),^7^,^9^ adverse physical and mental health (headaches, anxiety, and poor mental health outcomes related to problematic smartphone use, (such as anxiety, insomnia, increased perceived stress, poor educational attainment and decreased overall quality of life),^10^, ^11^, ^12^ and eye related correlates (such as dry eye syndrome, heavy eyelids, and increased myopia).^13^, ^14^, ^15^, ^16^ Furthermore, a systematic review has reported consistent associations between smartphone and/or tablet use and several measures of sleep outcomes in children, including significant associations between device use and poor sleep quality, quantity, and excessive daytime sleepiness.^17^ Despite these negative associations, however, problematic screen time behaviours are not consistently reported in studies measuring screen time, with authors arguing that problematic screen time and screen time should be measured concurrently using well established tools, such as the Smartphone Addiction Scale.^18^^,^^19^ In view of these reported associations between screen time and various negative outcomes, it is important to understand the effect that COVID-19 pandemic has had on screen time behaviours and how this has impacted on different correlates. To date, these changes in screen time during the pandemic and correlates have not been systematically reviewed and discussed. It was therefore the aim of this review to:

1. Aggregate and report on the changes in screen time in adults and children independently.
2. Report on all correlates that have been measured in relation to screen time during the COVID-19 pandemic.

This study has the potential to inform public health policy and guidance regarding screen time, and to inform future research in this area.

## Methods

### Protocol and registration

This systematic review was conducted in accordance with the Preferred Reporting Items for Systematic Reviews and Meta-Analyses (PRISMA) guidelines,^20^ and has been registered with the international prospective register of systematic reviews (PROSPERO protocol ID CRD42021261422). Note that there were no deviations from the published protocol.

### Search strategy

Databases were searched from inception to 06/12/2021 including Pubmed; Embase; Scopus; OpenGrey; Psycinfo; ERIC; Child Development and Adolescent Studies; and Web of Science, using the following search terms:

(screen time OR sedentary behaviour OR digital screen time OR sitting time OR screen-time OR gaming OR television OR smartphone OR computer time)

AND

(SARSCoV-2 OR 2019-nCoV OR COVID-19 OR coronavirus OR COVID19 OR coronavirus 19)

No other limiters were applied.

Results of searches were imported in a bibliographic database, with duplicates removed automatically. Titles and abstracts of the remaining studies were independently screened for inclusion by two authors (MT; EI). Following title and abstract screening, the full texts of all potentially eligible papers were reviewed independently by two reviewers (MT,EI) before making a final decision on eligibility, with a senior reviewer (SP) mediating any disputes. The following section describes the inclusion and exclusion criteria:

#### Population

All types of population (e.g., age, country) were considered, using any study design.

#### Intervention(s)/exposure(s)

All studies that reported pre vs during screen time usage during COVID-19, or studies that reported associations between any outcome and screen time during COVID-19.

#### Comparator(s)/control(s)

In studies that measured pre vs during COVID-19 screen time usage, the pre COVID-19 screen time data must have been collected prior to November 2019. Furthermore, all data were stratified into two group: adults (>18yrs), and children (<18yrs). Children were also stratified into three sub-groups were available: adolescents (11–17years), primary aged children (6–10years), and young children (<5 years).

#### Outcomes

Studies had to report one or more of the following:

- Mean screen time (in either hours or minutes/week) prior to and during the COVID-19 pandemic
- Percentage change (in terms of increased, remained the same, or decreased) screen time during the COVID-19 pandemic
- Associations between any outcome and screen time during the COVID-19 pandemic.

Exclusion criteria:

1. Written in languages other than English, Italian, French, or Spanish
2. Not been through the peer-review process (for example, pre-prints).

### Data extraction

Data were extracted by two reviewers (MT & RD) and included: first author; study title; publication date; country; study type; outcome type; outcome effect size; sample size; and participant characteristics.

### Risk of bias assessment

Risk of bias was assessed by two independent researchers (MT; EI) using the Newcastle Ottawa Scale (NOS) for cross-sectional studies.^21^^,^^22^ There are 3 parts in which studies are assessed and stars awarded: (i) selection (max. 5 stars) - representativeness of the sample, sample size, non-respondents, and ascertainment of the exposure (risk factor); (ii) comparability (max. 2 stars) - participants in different outcome groups are comparable; (iii) outcome (max. 3 stars)- assessment of outcome, and statistical test. Scores can range from 0 to 10 stars, with higher scores indicating better quality research. Any discrepancies over the final risk of bias verdict were solved by consensus, with involvement of a third review author (SP) where necessary.

### Statistical analysis

To aggregate screen time changes pre vs during COVID-19, a random-effects meta-analysis was conducted using the DerSimonian and Laird method, with studies weighted according the inverse variance, using Comprehensive Meta-Analysis.^23^ The meta-analysis was conducted using the following steps:

(1) Pre and during COVID-19 screen time (hrs/wk.), standard deviations were imputed, and means differences with standard errors were calculated. Note all analyses were stratified as adults (>18) and children (<18). Children were further stratified into three groups: (a) Adolescents (age 11–17); (b) children (age 6–10); and (c) young children (age 0–5). Note that only studies of the same study design were pooled.
(2) Heterogeneity between studies was assessed using the I² statistic,^24^ with 0–50% being considered low, 51–75% moderate, and >75% being considered high heterogeneity. If pooled results showed high heterogeneity, sub-group analysis was used to find the potential sources.
(3) Publication bias was assessed with a visual inspection of funnel plots and with the Egger bias test.^25^ As per the recommendations by Fu et al.^26^ and Sterne et al.,^27^ these tests were only conducted if the number of studies in each analysis exceeded ten. If significant publication bias (Egger's *p*=<0.05) was present, a trim-and-fill analysis was conducted.^28^
(4) Furthermore, sensitivity analyses were conducted to assess the robustness of analyses through the one study removed method.

Mean percent changes in screen time (increased, remain constant, and decreased) and SDs were also calculated using a random effects model, with studies weighted based on the inverse of the variance.

Due to anticipated heterogeneity in measurement types, all associations were aggregated in a narrative synthesis.

### Certainty of evidence

To ascertain the certainty of the evidence, the Grading of Recommendations, Assessment, Development and Evaluations^29^ (GRADE) framework was used.

### Role of the funding source

No funding was received for this study. All authors confirm that they had full access to all the data in the study and accept responsibility to submit for publication.

## Results

**Figure 1:**
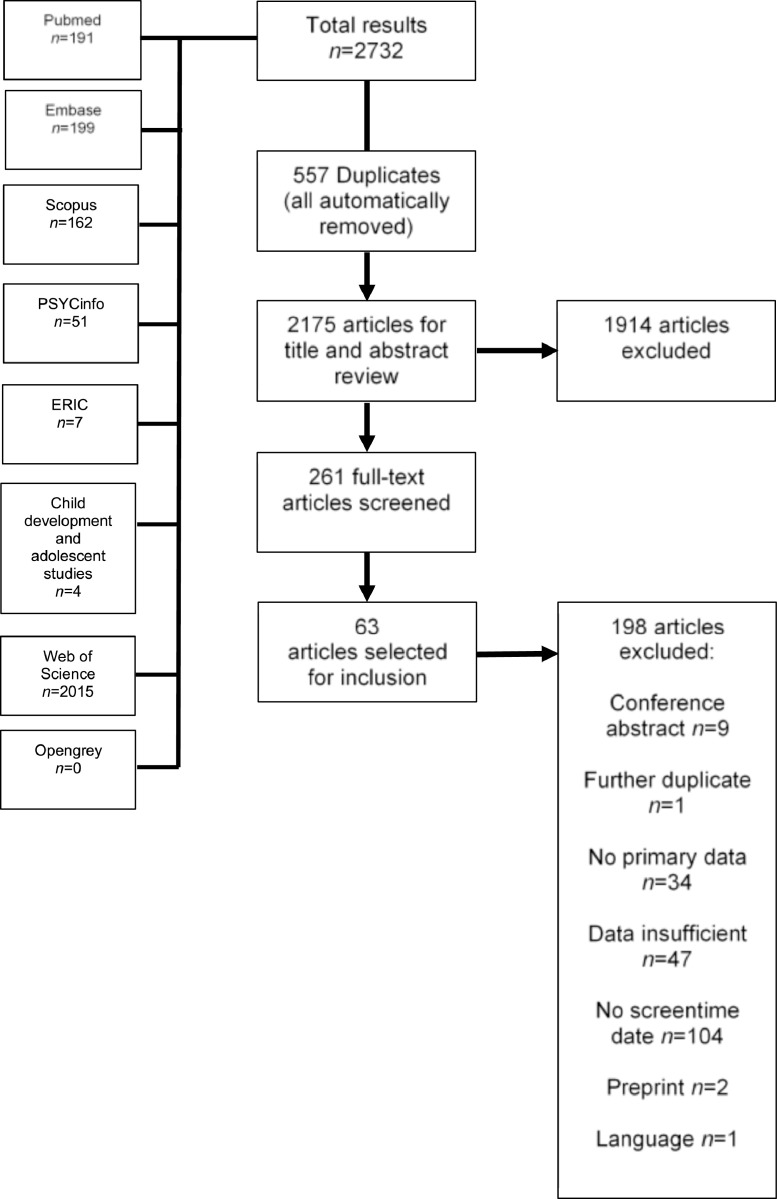
PRISMA flow diagram of study selection. Caption: PRISMA flow diagram showing the process of study selection. ERIC= Education Resources Information Centre.

The initial search yielded 7283 results, of which 1403 were removed as duplicates, leaving 5880 articles to be screened at the title and abstract level. Of these, 408 were selected for full text screening. After the full text assessment, 89 articles were selected for inclusion (total *n* 204,734; median age=20·6; median percentage female=53·3%). All but one of the included studies were cross-sectional in study design, with the one study being longitudinal. The full PRISMA diagram is shown in Figure 1. Of these, 46 studies included data regarding adults,^7^^,^^8^^,^^10^^,^^13^^,^^14^^,^^30^, ^31^, ^32^, ^33^, ^34^, ^35^, ^36^, ^37^, ^38^, ^39^, ^40^, ^41^, ^42^, ^43^, ^44^, ^45^, ^46^, ^47^, ^48^, ^49^, ^50^, ^51^, ^52^, ^53^, ^54^, ^55^, ^56^, ^57^, ^58^, ^59^, ^60^, ^61^, ^62^, ^63^, ^64^, ^65^, ^66^, ^67^, ^68^, ^69^, ^70^ and 46 studies included children.^9^^,^^11^^,^^15^^,^^42^^,^^66^^,^^69^^,^^71^, ^72^, ^73^, ^74^, ^75^, ^76^, ^77^, ^78^, ^79^, ^80^, ^81^, ^82^, ^83^, ^84^, ^85^, ^86^, ^87^, ^88^, ^89^, ^90^, ^91^, ^92^^,^^92^, ^93^, ^94^, ^95^, ^96^, ^97^, ^98^, ^99^, ^100^, ^101^, ^102^, ^103^, ^104^, ^105^, ^106^, ^107^, ^108^, ^109^ Furthermore, 32 studies^34^^,^^38^^,^^43^^,^^46^^,^^46^, ^47^, ^48^, ^49^^,^^51^^,^^52^^,^^55^, ^56^, ^57^^,^^65^^,^^68^^,^^70^, ^71^, ^72^, ^73^^,^^75^^,^^79^^,^^89^^,^^90^^,^^92^, ^93^, ^94^, ^95^^,^^98^^,^^100^, ^101^, ^102^^,^^108^ reported pre and during COVID-19 screen time data (and were included in the meta-analysis), 22 studies^32^^,^^35^^,^^37^^,^^39^^,^^42^^,^ ^45^^,^^54^^,^^56^^,^^59^^,^^61^^,^^66^^,^^67^^,^^77^^,^^78^^,^ ^80^^,^^82^^,^^84^^,^^85^^,^^87^^,^^96^^,^^97^^,^^104^ reported percentage change in screen time use during the pandemic, and 53 studies reported associations between several correlates and screen time use. Full descriptive characteristics are shown in Table 1. The mean NOS score was 6·4 (SD=0·9; range 4–8; see Supplementary Table 1 for full scoring information).

**Table 1:** Descriptive characteristics of included studies.

Author(s)	Country	Study design	Population type	Mean age (SD)	Age range	Percent female	Total n	Type of screen-time	Screen time reporting type	Risk of bias score
Abdulsalam et al.^30^	Saudi Arabia	Cross-sectional	Adults	NR	18–59	68%	472	Overall screen time	Online survey; self-report	4
Abid et al.^71^	Tunisia	Cross-sectional	Children	8·7 (3·3)	5–12	48%	100	Diurnal screen time; nocturnal screen time; global screen time	Online survey; self-report	8
Aguilar-Farias et al.^72^	Chile	Cross-sectional	Children	3·1 (1·4)	1–5	49%	3157	Overall screen time	Online survey; self-report	7
Agurto et al.^31^	Peru	Cross-sectional	Adults	NR	NR	NR	201	Sitting or lying in front of a screen	Online survey; self-report	7
Alomari et al.^32^	Jordan	Cross-sectional	Adults	33·7 (11·3)	18–72	70%	1844	Use of electronic screens; social media use; television use	Online survey; self-report	7
Alves et al.^11^	USA	Cross-sectional	Children (overweight or obese)	11·7 (1·2)	NR	57%	30	Leisure screen time	Telephone or video calls; parental self-report	7
			Children (healthy weight)	11·9 (1·2)	NR	68%	34	Leisure screen time		7
Balsam^14^	Saudi Arabia	Cross-sectional	Adults	33·4 (12·2)	18–81	72%	1939	Smartphone usage; daily digital device usage	Online survey; self-report	5
Beck et al.^73^	USA	Cross-sectional	Children	NR	4–12	55%	145	Non-academic screen time	Survey; parental report	7
Bird et al.^33^	UK	Cross-sectional	Adults	NR	18–85	80%	392	Overall screen time	Online survey; self-report	4
Branquinho et al.^34^	Portugal	Cross-sectional	Adults	48.5 (14.3)	NR	67.7%	5746	TV use; mobile phone use; social networking; gaming	Online survey; self-report	6
Breidokiene et al.^74^	Lithuania	Cross-sectional	Children	9.7 (1.9)	6–14	52.9%	306	Screentime for leisure; screen time for education	Online survey; parental report	6
Brzek et al.^75^	Poland	Cross-sectional	Children	NR	3–5	NR	1311	TV use; tablet time; PC time; mobile time	Online survey; parental report	6
Cachon-Zagalaz et al.^76^	Spain	Cross-sectional	Children	6·2 (3·4)	0–12	50%	837	Daily use of digital screens	Online survey; self-report	7
Cahal et al.^77^	Israel	Cross-sectional	Children	6·2 (4·7)	0–18	38%	445	Overall screen time	Online survey; self-report	7
Chambonniere et al.^78^	France	Cross-sectional	Children	NR	6–10	46%	1588	Overall screen time	Online survey; self-report	7
			Children	NR	11–17	61%	4903	Overall screen time		7
Cheikh Ismail et al.^35^	UAE	Cross-sectional	Adults	NR	NR	76%	1012	Screen time for entertainment	Online survey; self-report	6
Chen et al.^79^	China	Longitudinal	Children	10.3 (0.8)	NR	50.5%	535	Smartphone use	Online survey; self-report	7
Conroy et al.^36^	USA	Cross-sectional	Adults	43 (13)	NR	79%	834	Screentime before bed	Online survey; self-report	5
Constandt et al.^110^	France	Cross-sectional	Adults	NR	NR	51.2%	4005	Overall screen time	Online survey; self-report	6
Coyne et al.^38^	Canada	Cross-sectional	Adults	39.2 (15.1)	21–77	87%	64	Recreational screen time	Online survey; self-report	7
de Sa et al.^80^	Brazil	Cross-sectional	Children	NR	0–12	NR	816	Playful screen time	Online survey; parental report	6
Donati et al.^81^	Italy	Cross-sectional	Children	11.1 (NR)	NR	27%	554	Gaming	Online survey; parental and child report (not stratified)	5
Dragun et al.^39^	Croatia	Cross-sectional	Adults (international medical students)	22 (6)	NR	63%	59	Computer/table use time; television watching time; mobile use time	Online survey; self-report	7
			Adults (domestic medical students)	23 (6)	NR	78%	148	Computer/table use time; television watching time; mobile use time		4
Dubuc et al.^82^	Canada	Cross-sectional	Children	NR	NR	53.3%	2661	Recreational screen time	Online survey; self-report	7
Farah et al.^83^	Israel	Cross-sectional	Children	2 (0·6)	1–3	47%	NR	Screen exposure	Online survey; parental report	6
Fillon et al.^84^	France	Cross-sectional	Children	NR	1–6	49.7%	348	Overall screen time	Online survey; parental report	5
Fraser et al.^40^^(p19)^	USA	Cross-sectional	Adults (college students)	NR	NR	70%	74	TV use; social media; gaming	Online survey; self-report	7
Ganne et al.^41^	India	Cross-sectional	Adults	23.4 (8.2)	18–79	48.9%	941	Overall screen time	Online survey; self-report	6
Garcia et al.^85^	USA	Cross-sectional	Children with autism	NR	NR	11%	9	Overall screen time; weekday screen time	Online survey; self-report	6
Genin et al.^42^	France	Cross-sectional	Children	NR	6–10	NR	1588	Overall screen time	Online survey; self-report	8
			Children	NR	11–17	NR	4903	Overall screen time		7
			Adults	NR	18–64	NR	15,226	Overall screen time		7
			Adults	NR	65+	NR	1178	Overall screen time		6
Giannini et al.^86^	Brazil	Cross-sectional	Children	15.3 (1.8)	12–18	57.7%	208	Overall screen time	Online survey; self-report	7
Gornika et al.^7^	Poland	Cross-sectional	Adults	NR	NR	89.8%	2381	Overall screen time	Online survey; self-report	7
Guo et al.^87^^(p19)^	China	Cross-sectional	Children (primary, secondary, and high school students)	Median=13 (IQR=10–16)	NR	49.9%	10,416	Overall screen time	Online survey; self-report	7
Hadianfard et al.^88^	Iran	Cross-sectional	Children	NR	12–16	49.4%	510	Overall screen time	Online survey; self-report	7
Hashem et al.^9^	Egypt	Cross-sectional	Children	NR	4–16	47%	765	Electronics and screen use; mobile extra screen time; television extra screen time; laptop extra screen time	Online survey; self-report	6
Helbach and Stahlmann^43^	Germany	Cross-sectional	Adults	22.4 (2)	18–26	76%	884	Smartphone use; TV use; PC/computer/tablet use	Online survey; self-report	8
Hodes et al.^44^	South Africa	Cross-sectional	Adults	20·5 (1·5)	18–25	NR	244	Objectively measured smartphone use	iPhone screen time data shared via screenshots	6
Hu et al.^45^	China	Cross-sectional	Adults	NR	18–60	48%	1033	Leisure screen time; overall screen use	Online survey; self-report	7
Hyunshik et al.^89^	Japan	Cross-sectional	Children	4.8 (0.3)	3–5	47.8%	290	Overall screen time	Online survey; parental report	6
Jáuregui et al.^90^	Mexico	Cross-sectional	Children	3.3 (NR)	1–5	47.2%	631	Overall screen time	Online survey; parental report	6
Jia et al.^46^	China	Cross-sectional	Adults (undergraduate students)	20.6 (1.8)	NR	70%	7024	Overall screen time	Online survey; self-report	6
			Adults (graduate students)	24.6 (3.5)	NR	71%	234	Overall screen time	Online survey; self-report	7
Kim et al.^91^	South Korea	Cross-sectional	Children	9.2 (1.4)	7–12	43.8%	217	TV time; tablet time; smartphone time	Online survey; parental report	6
Koohsari et al.^47^	Japan	Cross-sectional	Adults	NR	NR	NR	1086	Television time; PC use during workday; PC use sitting time	Online survey; self-report	6
Kowalsky et al.^48^	USA	Cross-sectional	Adults	22.1 (4.9)	NR	73%	189	TV/computer/phone use	Online survey; self-report	5
Lawrence et al.^49^	USA	Cross-sectional	Adults (social care students)	29 (10)	NR	93%	88	Overall screen time	Online survey; self-report	7
Le et al.^50^	USA	Cross-sectional	Adtuls (healthcare workers)	NR	25–64	51%	74	Overall screen time	Online survey; self-report	7
Lim et al.^92^	Singapore	Cross-sectional	Children	Median 8 (IQR 6–11)	NR	NR	593	Non-academic screen time	Online survey; parental report	7
Liu et al.a^111^	China	Cross-sectional	Children	NR	NR	48%	3405	e-learning screen use	Online survey; self-report	6
Lui et al. b^15^	China	Cross-sectional	Children	NR	NR	48%	3831	Non-academic screen time	Online survey; self-report	6
Lopez-Gil et al.^93^	Spain	Cross-sectional	Children	NR	3–17	50%	604	Sedentary screen-based pursuits	Online survey; parental report	6
	Brazil		Children		3–17	44%	495			5
Ma et al.^94^	China	Cross-sectional	Children	8.9 (0.7)	8–10	47.6	208	Digital screen time(not including online education)	Online survey; self and parental report	6
Majumdar et al.^51^	India	Cross-sectional	Adults (office workers)	33·1 (7·1)	NR	18%	203	Cell phone use; desktop/laptop use; television use	Online survey; self-report	5
			Adults (students)	22·1 (1·7)	NR	61%	325			7
McArthur et al.^95^	Canada	Cross-sectional	Children	9·9 (0·8)	8–9·5	48%	1333	Screen time reported by child; screen time reported by mother	Online survey; parental report	6
McCormack et al.^96^	Canada	Cross-sectional	Children	10.8 (4)	NR	45.1%	328	Use of screen based devices; TV use; gaming	Online survey; self-report	7
Meyer et al.^10^	USA	Cross-sectional	Adults	NR	NR	NR	1540	Overall screen time	Online survey; self-report	7
Mitra et al.^97^	Canada	Cross-sectional	Children	NR	5–11	NR	693	Overall screen time	Online survey; parental report	7
			Children	NR	12–17	NR	779	Overall screen time		8
Mohan et al.^98^	India	Cross-sectional	Children	13 (2·5)	10–18	54%	261	Overall screen time	Online survey; parental and self-report	7
Mon-Lopez et al.^52^	Spain	Cross-sectional	Adults	39.7 (13.6)	NR	50%	120	Overall screen time	Online survey; self-report	4
Nassar et al.^99^	Egypt	Cross-sectional	Children (soccer players)	NR	9–11	0%	74	Overall screen time	Online survey; self-report	6
Nathan et al.^100^	Australia	Cross-sectional	Children	6.9 (1.7)	5–9	45.9%	157	Leisure screen time	Online survey; self-report	5
Oswald et al.^53^	Australia	Cross-sectional	Adults	21·2 (1·9)	18–24	55%	55	Overall screen time	Online survey; self-report	7
Pavithra and Sundar^13^	India	Cross-sectional	Adults (engineering students)	NR	NR	54%	396	Overall screen time	Online survey; self-report	7
Peddie et al.^101^	New Zealand	Cross-sectional	Children (adolescent boys)	16.6 (0.7)	15–18	0%	109	Overall screen time	Online survey; self-report	7
Robbins et al.^54^	USA	Cross-sectional	Adults (elderly adults)	NR	65+	NR	3122	TV time	Online survey; self-report	6
Rodriguez-Larrad et al.^55^	Spain	Cross-sectional	Adults	22·8 (5·3)	18–54	65%	13,754	Leisure screen time; study screen time	Online survey; self-report	6
Sallie et al.^56^	International cohort	Cross-sectional	Adults	28.9 (12.5)	18–90	24.2%	771	Online gaming	Online survey; self-report	7
							859	Porn viewing		6
Sanudo et al.^70^	Spain	Cross-sectional	Adults	22·6 (3·4)	NR	45%	20	Overall screen time	Phone usage data; objectively measured	8
Saxena et al.^57^	India	Cross-sectional	Adults (college students)	20·4 (1·4)	18–24	50%	60	Overall screen time	Online survey; self-report	5
Schmidt et al.^102^	Germany	Cross-sectional	Children	NR	4–17	NR	1711	Television use; gaming time; recreational internet use; recreational screen time	Online survey; self-report	6
Sewall et al.^58^	USA	Cross-sectional	Adults	24·5 (5·1)	18–35	57%	384	Objectively measured screen time	iPhone screen time data shared via screenshots	7
Sikorska et al.^103^	International cohort	Cross-sectional	Children	NR	11–16	65.7%	370	Online gaming; internet browsing; TV use; social media	Online survey; self-report	7
Siste et al.^104^	Indonesia	Cross-sectional	Children	17.4 (2.2)	NR	78.7%	2932	Internet duration	Online survey; self-report	6
Spence et al.^59^	UK	Cross-sectional	Adults	NR	NR	51%	1521	Overall screen time; screen time for work/school; screen time for leisure	Online survey; self-report	6
Stieger et al.^60^	Austria	Cross-sectional	Adults	31 (14·5)	NR	56%	286	Overall screen time	App-collected; self-report	7
Stokes et al.^105^	Australia	Cross-sectional	Children	10.6 (3.1)	5–17	23.6%	213	TV use; social media use; gaming	Online survey; self-report	7
Suka et al.^61^	Japan	Cross-sectional	Adults	NR	25–64	NR	8000	Television use; digital media exposure	Online survey; self-report	6
Szwarcwald et al.^106^	Brazil	Cross-sectional	Children	NR	12–17	50%	9470	Overall screen time	Online survey; self-report	6
Tan et al.^62^	Malaysia	Cross-sectional	Adults (university students)	22 (2.3)	18–27	74.2%	186	Sedentary screen time	Online survey; self-report	6
Tebar et al.^8^	Brazil	Cross-sectional	Adults	37·9 (13·3)	NR	59%	1896	Television use; cell phone use; computer time	Online survey; self-report	4
Werneck et al.^64^	Brazil	Cross-sectional	Adults	NR	NR	53%	33,862	Television use; computer/tablet use	Online survey; self-report	8
Windiani et al.^107^	Indonesia	Cross-sectional	Children	Median 16	15–18	49%	204	Overall screen time	Online survey; self-report	7
Woodruff et al.^65^	Canada	Cross-sectional	Adults	36·2 (13·1)	18–77	80%	80	Screen related sedentary behaviour	Online survey; self-report	7
Wunsch et al.^108^	Germany	Cross-sectional	Children	NR	4–17	NR	1686	Overall screen time	Online survey; self-report	7
Xiao et al.^109^	China	Cross-sectional	Children	NR	NR	49%	1680	Online study time; other screen time	Online survey; self-report	7
Yang et al.^66^†	China	Cross-sectional	Adults (graduate students)	24·6 (3·5)	NR	71%	234	Overall screen time	Online survey; self-report	7
			Adults (undergraduate students)	20·6 (1·6)	NR	70%	7024	Overall screen time		5
			Children (high school students)	17·5 (1·2)	NR	76%	2824	Overall screen time		7
Zajacova et al.^67^	Canada	Cross-sectional	Adults	NR	NR	51%	4319	Internet time; television time	Online survey; self-report	4
Zarco-Alpeunte et al.^63^	Spain	Cross-sectional	Adults	NR	18–55	NR	886	TV use; online sexual activities; video games; social networks; online shopping; instant messaging	Online survey; self-report	6
Zhang et al.^68^	China	Cross-sectional	Adults (pregnant women)	29 (4)	NR	100%	1794	Overall screen time	Online survey; self-report	6
Zhou et al.^69^†	China	Cross-sectional	Adults (graduate students)	24·6 (3·5)	NR	71%	234	Overall screen time	Online survey; self-report	6
			Adults (undergraduate students)	20·6 (1·6)	NR	70%	7024	Overall screen time		7
			Children (high school students)	17·5 (1·2)	NR	76%	2824	Overall screen time		7

† Yang et al. and Zhou et al. used the same sample, with data reported in different formats.

**Table 2:** Meta-analytic changes in any type of screen time in hrs/day, stratified between adults and children.

Population		*n* studies(*k* outcomes)	*n* participants	Pooled screen time change(95%CI)	p-value	I^2^	Eggers's bias(p-value)	Trim and fill adjustment(95%CI; n studies trimmed)
Total screen time	Adults	13 (33)	30,514	0·96 (0·70–1·22)	<0·001	99·80	0·10 (1·00)	NA
	Adolescents	8 (21)	6495	0·91 (0·32–1·50)	0·003	99·96	−4·56 (0·80)	NA
	Primary aged children	11 (21)	5566	1·39 (1·10–1·69)	<0·001	99·76	3·24 (0·69)	NA
	Young children	7 (25)	5991	0·59 (0·29–0·91)	<0·001	99·91	−32·17 (<0·001)	0·70 (0·43–0·97; 4 studies)
Leisure screen time	Adults	7 (15)	22,921	0·72 (0·29–1·15)	0·001	99·89	−3·36 (0·84)	NA
	Adolescents	3 (10)	2102	0·48 (0·29–0·67)	<0·001	98·14	11·02 (0·04)	0·61 (0·31–0·90; 2 studies)
	Primary aged children	6 (10)	2202	1·04 (0·77–1·30)	<0·001	99·03	15·78 (<0·001)	1·12 (0·70–1·54; 1 study)
	Young children	3 (8)	1767	0·61 (0·40–0·82)	<0·001	98·78	NA	NA

### Meta-analytic changes in screen time

**Figure 2:**
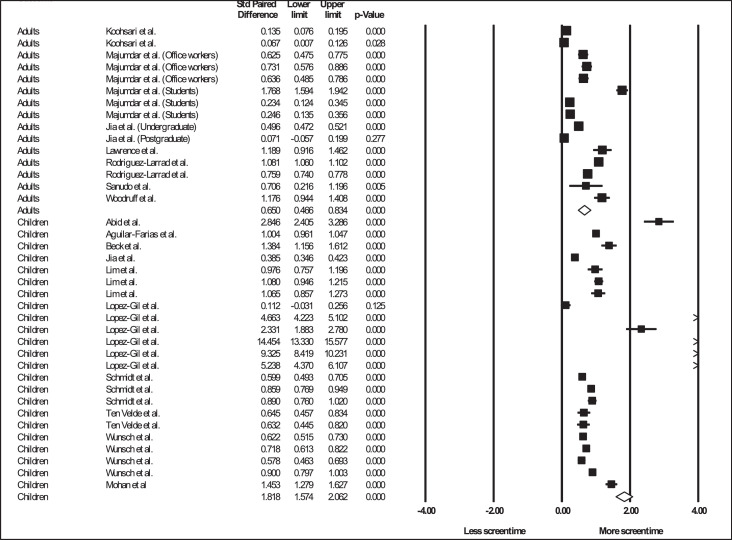
Forest plot showing pooled changes in any type of screentime from before the COVID-19 pandemic, stratified by adults or children. Caption: Units=hrs/day; Error bars= 95% confidence interval; Solid boxes = individual study point estimates; Clear box = Pooled point estimates.

There were 133 outcomes yielded from 32 studies in the meta-analysis. Regarding total screen time (see Figure 2), adults reported increases of 0·96 hrs/day (95%CI 0·70–1·22 hrs/day; I^2^=99·80; *k* = 33), adolescents 0·91 hrs/day (95%CI 0·32–1·50; I^2^=99·96; *k* = 21), primary aged children 1·39 hrs/day (95%CI 1·1–1·69; I^2^=99·76; *k* = 21), and young children 0·59 hrs/day (95%CI 0·29–0·91; I^2^=99·91; *k* = 25). The analysis of total screen time in young children showed significant publication bias (Egger's *p*=<0.001). Subsequent trim and fill analysis yielded a significant increase of 0·70 hrs/day (95%CI 0·43–0·97), with four studies trimmed to the right of the mean. The magnitude or direction of results were not influenced by the removal of any one study. Because all analyses had high heterogeneity, all these results were classified as of ‘very low’ certainty according to the GRADE criteria.

**Figure 3:**
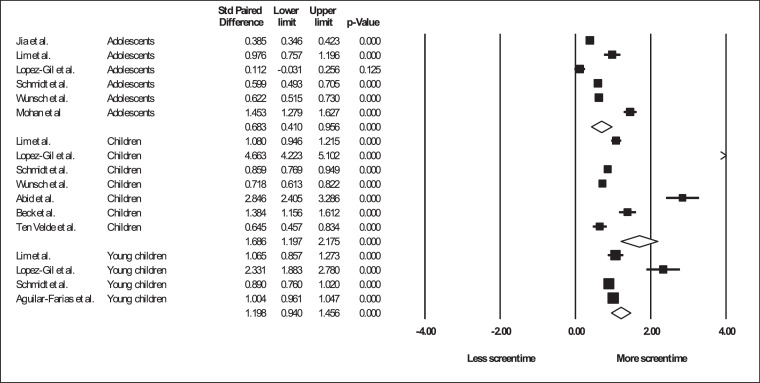
Forest plot showing pooled changes in any type of screentime from before the COVID-19 pandemic in children, stratified by age group. Caption: Units=hrs/day; Error bars= 95% confidence interval; Solid boxes = individual study point estimates; Clear box = Pooled point estimates.

In studies that reported changes in leisure screen time (non-work/non-academic; see Figure 3), adults reported increases of 0·72 hrs/day (95%CI 0·29–1·15 hrs/day; I^2^=99·89; *k* = 15), adolescents 0·48 hrs/day (95%CI 0·29–0·67; I^2^=98·14; *k* = 10), primary aged children 1·04 hrs/day (95%CI 0·77–1·30; I^2^=99·03; *k* *=* 10), and young children 0·61 hrs/day (95%CI 0·40–0·82; I^2^=98·78; *k* = 8). The analysis of leisure screen time in adolescents and primary aged children showed significant publication bias (Egger's *p* = 0.04 and <0.001, respectively), with the subsequent trim and fill analyses yielding respective significant increase of 0·61 hrs/day (95%CI 0·31–0·90; two studies removed) and 1·12 hrs/day (95%CI 0·70–1·54; one study removed), with all studies trimmed to the right of the mean. The magnitude or direction of results were not influenced by the removal of any one study. Due to the study design (all included studies were cross-sectional) and high heterogeneity, all these results were classified as ‘very low’ certainty according to the GRADE criteria.

### Percent changes in screen time

**Figure 4:**
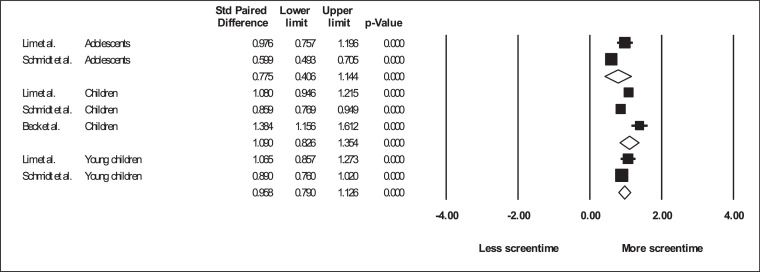
Forest plot showing pooled changes in leisure screentime from before the COVID-19 pandemic in children, stratified by age group. Caption: Units=hrs/day; Error bars= 95% confidence interval; Solid boxes = individual study point estimates; Clear box = Pooled point estimates.

**Figure 5:**
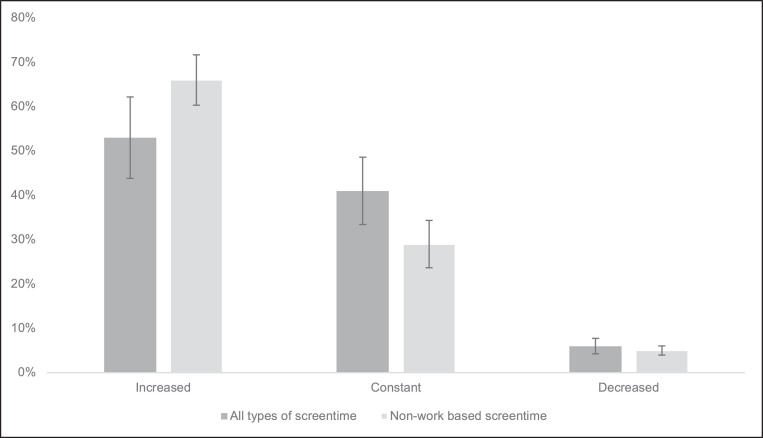
Percent changes in screentime in adults. Caption: Error bars show 95% confidence intervals.

As shown in Table 3 and Figure 4, the random effects model yielded 51% (95%CI 44–58) of adults reporting an increase in total screen time, 39% (95% CI 33–46) no change, and 7% (95% CI 5–9) a decrease. Regarding leisure screen time (non-academic or non-work related), 52% (95% CI 38–66) of adults reported an increase, 38% (95% CI 27–51) reported no change, and 7% (95% CI 5–9) reported a decrease. In children, 67% (95% CI 60–74) reported an increase in total screen time, 27% (95% CI 21–33) reported no change, and 4% (95% CI 3–6) reported a decrease. Regarding leisure (non-academic or non-work related) screen time, 59% (95% CI 50–69) of children reported an increase, 30% (95% CI 24–35) reported no change, and 9% (95% CI 6–14) reported a decrease (see Figure 5). Stratification of children into age-groups was not possible due to a paucity of data.

**Table 3:** Pooled changes in screen time over the COVID-19 pandemic reported as percentages.

		*n* studies(*k* outcomes)	*n* participants	Increased(95% CI)	No change(95% CI)	Decrease(95% CI)
Total screen time	Adults	13 (26)	59,405	50·8% (44·0–57·6%)	39·0% (32·9–45·5%)	7·0% (5·1–9·4%)
	Children	12 (21)	34,467	67·3% (59·8–74%)	26·8% (21·1–33·3%)	4·0% (2·7–6·0%)
Leisure screen time (non-work/non-academic)	Adults	4 (6)	6733	52·4% (38·3–66·2%)	38·0% (26·8–50·5%)	6·7% (4·8–9·3%)
	Children	3 (6)	3805	59·4% (49·6–68·5%)	29·5% (24·2–35·3%)	9·0% (5·7–13·9%)

**Figure 6:**
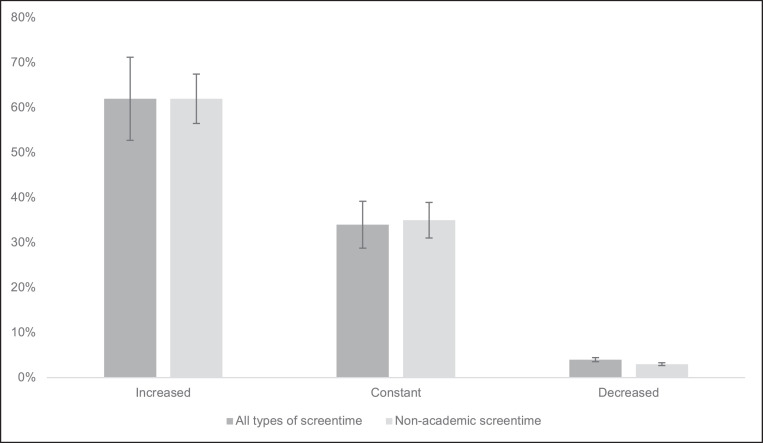
Percent changes in screentime in children Caption: Error bars show 95% confidence intervals.

### Associations between screen time and multiple correlates

#### Adults

In adults, 30 studies^7^^,^^8^^,^^10^^,^^13^^,^^14^^,^^30^^,^^31^^,^^33^^,^^36^^,^^40^^,^^41^^,^^43^, ^44^, ^45^^,^^47^^,^^49^^,^^50^^,^^52^, ^53^, ^54^^,^^57^^,^^58^^,^^60^, ^61^, ^62^, ^63^, ^64^^,^^66^^,^^68^^,^^69^ reported 109 outcomes across the following areas: diet and smoking, eye health, mental health, fatigue, general health, physical activity, and weight gain/BMI (see Supplementary Table 2).

##### Diet and smoking

Three studies^7^^,^^8^^,^^62^ (yielding 29 independent outcomes) reported correlates in relation to diet and smoking, of which 35% (10/29) were statistically significant.

In outcomes that were related to overall screen time, 50% (2/4) of the outcomes yielded significant outcomes. The significant outcomes included a negative association between increases in screen time and a ‘constant diet’ during COVID (OR=0·68; 95%CI 0·56–0·82),^8^ and increases in screen time was reported to be associated with ‘unhealthy dietary changes’ (OR=1·54 95%CI 1·21–1·96).^8^ Non-significant associations were found between increases in screen time and ‘pro-healthy’ dietary changes, or ‘self-regulation around eating’.^7^

Regarding TV time, 43% (3/7) outcomes were statistically significant, including a significant negative correlation between TV time and self-regulation around eating (*r*= −0·24; *p* = 0·01),^62^ positive associations between increased TV use and increased desire to drink (OR=1·46 95%CI 1·12–1·89),^8^ and increases in sweetened food consumption (OR=1·53 95%CI 1·12–1·89).^8^ Non-significant findings included increased TV use and alcohol consumption, increased desire to smoke, and increases in smoking.

In outcomes that measured gaming, 50% (1/2) of univariate correlations were statistically significant. The significant association was a negative correlation between the use of gaming consoles and self-regulation around eating (*r*= −0·15; *p* = 0·04),^62^ and the non-significant association was ‘gaming on a computer’ and self-regulation around eating (*r*= −0·06; *p*=NR).^62^

Regarding cell phone use, 17% (1/6) of outcomes yielded significant associations, with increases in cell phone use being significantly associated with the consumption of sweetened foods (OR=1·78 95%CI 1·18–2·67).^8^ Increases in cell phone use was not significantly associated with alcohol consumption, increased desire to drink alcohol, increased in smoking or the desire to smoke more.

In outcomes that measured computer-based screen time (including internet use), 33% (3/9) of outcomes were significant. Of these significant outcomes, increases in computer time was negatively associated with alcohol (OR=0·68; 95%CI 0·53–0·86), and sweetened foods (OR=0·78; 95%CI 0·62–0·98)^8^ consumption. Furthermore, internet use for self-directed learning was found to be positively associated with self-regulation around eating in univariate analyses (*r* = 0·17; *p* = 0·02).^62^

##### Eye health

Three studies^13^^,^^14^^,^^41^ (yielding 17 independent outcomes) examined associations between screen time and eye health, 88% (15/17) being statistically significant. Regarding the type of screen time, all studies examined increases in total screen time. All three studies that measured dry eye syndrome found significant positive associations (increased screen time OR=66·7 95%CI 20·4–218·3; <6 hrs/day with >6 hrs/day as the reference group; OR 0·51 95%CI 0·39–0·67; *x*^2^=39.2 *p*=<0.001).^13^^,^^14^^,^^41^ The remaining associations measured symptoms of digital eye strain,^14^ and found significant associations between less than 6 hrs/day of screen time (with >6 hrs/day as the reference group) and tearing (OR=0·72 95%CI 0·54–0·96); eye strain (OR=0·51 95%CI 0·41–0·64); eye dryness (OR=0·62 95%CI 0·49–0·79); heavy eyelids (OR=0·68 95%CI 0·51–0·91); eye redness (OR=0·60 95%CI 0·44–0·81); eye itchiness (OR=0·53 95%CI 0·40–0·69); burning sensations in the eye (OR=0·59 95%CI 0·45–0·76); sensitivity to bright light (OR=0·58 95%CI 0·43–0·79); difficulty focussing (OR=0·70 95%CI 0·55–0·90); eye pain (OR=0·56 95%CI 0·41–0·75); foreign body sensation in the eye (OR=0·69 95%CI 0·49–0·98); and excessive blinking (OR=0·68 95%CI 0·52–0·87).^14^ Neither diplopia and blurred vision were associated with increases in screentime.^14^

##### Mental health

A total of twenty studies^10^^,^^14^^,^^33^^,^^36^^,^^40^^,^^44^^,^^45^^,^^47^^,^^49^^,^^50^^,^^52^, ^53^, ^54^^,^^58^^,^^60^^,^^63^^,^^64^^,^^66^^,^^68^^,^^69^ (yielding 68 independent outcomes) examined associations between screen time and mental health outcomes, with 46% (31/68) outcomes yielding significant results.

Seventeen outcomes measured screen time and anxiety, with 47% (8/17) of outcomes being statistically significant. Regarding increases in overall screen time, several nominal categories of screen time were associated with anxiety (with less than 2 hrs/day as the reference variable) with higher levels of screen time showing higher odds ratios: 5–6 hrs/day (OR=1·76 95%CI 1·20–2·58), 7–8 hrs/day (OR=1·98 95%CI 1·29–3·03), and more than 8 hrs/day (OR=2·22 95% 1·45–3·40), however 3–4 hrs/day was not significant.^68^ This is in agreement with two other studies who also found significant positive associations between overall screen time and anxiety (β=1·34; *p* = 0·003^10^ and β=0·93; *p* = 0·04^49^). Conversely, both studies that measured screen time objectively (using smartphone data) yielded no significant associations.^44^^,^^58^ Furthermore, Le et al.^50^ found no significant associations between screen time and anxiety. Increases in TV use was associated with anxiety about COVID-19 (prevalence ratio=1·4 95% CI 1·2–1·6), and overall anxiety in people with (OR=1·58; *p*=<0.05), and without depression (OR=1·73; *p*=<0.05).^64^ Concurrently, decreases in TV use yielded non-significant associations with anxiety about COVID-19,^54^ and anxiety in people with and without depression.^64^

Depression was measured in eight outcomes, with 63% (5/8) being significant, including increasing TV use and ‘depression about COVID-19′ (prevalence ratio=1·3 95%CI 1·1–1·5), with a concurrent non-significant association between decreases in TV use and ‘depression about COVID-19′.^54^ Overall screen time yielded conflicting results, with two studies^10^^,^^69^ reporting positive associations with depression (OR=1·54 95%CI 1·03–2·30; β=1·92; *p*=<0·001), another^68^ reporting that increases in screen time yield negative (protective) associations with depressive symptoms (OR=0·54 95%CI 0·43–0·65), and others yielding no significant results.^49^^,^^58^

Loneliness was examined in 15 outcomes, with 47% (7/15) of outcomes yielding significant results. Of the significant outcomes, overall screen time was reported to be associated with onliness in one study (β =0·34; *p*=<0·001).^10^ Social loneliness was associated with social media use (β of direct effect=0·54; *p*=<0·001; β of indirect effect= −0·01; *p*=<0·05),^66^ and internet gaming use (β of direct effect=0·43; *p*=<0·001; β of indirect effect was not significant).^66^ Emotional loneliness was associated with social media use (β of direct effect=0·52; *p*=<0·001; β indirect effect was not significant), and internet gaming use (β of direct effect=0·44; *p*=<0·001; β of indirect effect was not significant).^66^ Increases in TV use were also reported to be significantly associated with loneliness in people without depression (OR=1·59; *p*=<0.05), whereas this result was not significant in people with depression.^64^ The same study also found non-significant associations between decreases (and no changes) in screen time and loneliness in people with and without depression.^64^

Two studies^36^^,^^50^ examined mood changes and screen time, with both studies yielding non-significant results.

Regarding other aspects of mental health, studies report conflicting results. TV time (β=0·16; *p*=<0·001), online shopping (β=0·16; *p*=<0·01), and online sexual activities (β=0·13; *p*=<0·05) were all correlated with the impact of COVID-19 lockdowns on overall mental health, whereas total screen time, video gaming, social media use and instant messaging were non-significant.^33^^,^^63^ Positive affect was significantly negatively correlated with instant messaging (β=−0·1; *p*=<0·01) and TV use (β= −0·09; *p*=<0·05), but not significantly associated with online sexual activities, video games, social media use, or online shopping.^63^ Negative affect was significantly negatively associated with social media use (β= −0·08; *p*=<0·05), and positively associated with online shopping (β= 0·15; *p*=<0·05), but not with video games or instant messaging.^63^ Furthermore, increases in screen time were negatively associated with overall wellbeing in one study (ICC *r*= −0·31 *p*=<0·001),^60^ but not in others.^45^ ‘Struggling’ versus ‘flourishing’ mental health was associated with increases and decreases in screen time (RR=2·20; *p=<*0·05 and RR=23·85; *p=<*0·05 respectively), indicating widely conflicting results.^53^ Headaches were reported to be negatively associated with less than 6 hrs/day of screen time (OR=0·55; *p*=<0.001), and boredom was consistently associated with increases in social media use (β of direct effect=0·46; *p*=<0·001; β of indirect effect=0·03; *p*=<0·05) and internet gaming (β of direct effect=0·39; *p*=<0·001; β of indirect effect=0·04; *p*=<0·05).^66^

The remaining outcomes were non-significant, including leisure screen time and subjective wellbeing,^45^ increasing screen time and ‘languishing’ versus ‘flourishing’ mental health,^53^ and overall mental health.^33^ Furthermore, increases in instant messaging, social media use, and video games were all not significantly associated with COVID-19 related overall mental health.^63^

##### Sleep/fatigue

Two studies^47^^,^^50^ yielded 14 independent outcomes regarding sleep/fatigue and concentration, all of which were not significant.

##### General health

One study^61^ examined screen time and general health, with two outcomes. Of these 50% (1/2) were significant. Digital media exposure was significantly associated with general health (OR=1·14 95%CI 1·03–1·27), and TV use was not.^61^

##### Physical activity

Three studies^30^^,^^43^^,^^47^ yielding 10 independent outcomes reported associations between screen time and physical activity, with 50% (5/10) being significant. Associations between overall screen time and physical activity consistently yielded significant results (β= −0·08; *p*=<0·001; One study^30^ did not report an effect size, but reported that they had a significant association).^30^^,^^43^ Conflicting results were found regarding TV use and physical activity, with one study reporting significant associations (β= −0·15; *p*=<0·01),^43^ and another showing no significant associations.^47^ Significant associations were found between gaming (β= −0·21; *p* = 0·04),^43^ and social media use (β= −0·06; *p* = 0·04),^43^ but not smartphone use,^43^ or PC/computer/tablet use.^43^^,^^47^

##### Weight gain/BMI

There were two studies,^31^^,^^57^ each reporting one outcome each, regarding weight gain. One study found that time spent lying in front of a screen was significantly higher in people who had gained weight during COVID-19, compared to people whose weight had stayed consistent or lost weight (*X*^2^ NR^;^ *p* = 0·002),^31^ with the other study reporting no differences between overall screen time and BMI.^57^

#### Children

In children, 24 studies^9^^,^^11^^,^^15^^,^^66^^,^^72^^,^^74^^,^^76^^,^^79^^,^ ^81^^,^^83^^,^^86^^,^^88^^,^^90^, ^91^, ^92^^,^^95^^,^^98^^,^^99^^,^^103^^,^^105^, ^106^, ^107^^,^^109^^,^^111^ reported 181 outcomes across the following areas: diet, eye health, mental health, physical activity, parental health, physiology, sleep, and problematic behaviours (see Supplementary Table 3).

##### Diet

One study^9^ with 35 independent outcomes examined associations between screen time and diet, with 54% of outcomes (19/35) yielding significant results. ‘Extra mobile screen time’ was significantly associated with increases in: appetite (*r* = 0·13; *p*=<0·001), sweets and unhealthy food consumption (*r* = 0·07; *p* = 0·04), not caring about eating fruit and vegetables (*r* = 0·09; *p* = 0·01), late snacking at night (*r* = 0·16; *p*=<0·001), and decreases in regular protein intake (*r* = 0·11; *p* = 0·003). Non-significant associations included decreases in (or loss of) appetite, and frequently snacking between meals.

Extra TV time was significantly associated with increases in: frequent snacks between meals (*r* = 0·08; *p* = 0·04), late snacks at night (*r* = 0·09; *p* = 0·01), and decreases in (or loss of) appetite (*r* = 0·07; *p*=<0·05). Increases in appetite, not caring about eating fruits and vegetables, and decreases in protein intake were all not significantly associated with extra TV time.

‘Extra laptop screen time’ was positively associated with: not caring about eating fruit and vegetables (*r* = 0·10; *p* = 0·005), frequent snacking in between means (*r* = 0·20; *p*=<0·001), appetite (*r* = 0·16; *p*=<0·001), and negatively associated (a protective effect) with loss of appetite (*r*=−0·14; *p*=<0·001). Conversely, increases in sweets and unhealthy foods, decreases in protein intake, and snaking during the night were all not associated with extra laptop screen time.

Increases in ‘video gaming’ were associated with increases in appetite (*r* = 0·12; *p*=<0·001), and late snacking during the night (*r* = 0·09; *p* = 0·02). Non-significant findings included decreases (or loss of): appetite, protein intake, increases in sweets and unhealthy foods, not caring about eating fruits and vegetables, and frequent snacking between meals.

Remote learning was negatively associated (a protective effect) with increases in snacking in between meals (*r*=−0·08; *p* = 0·02), and positively associated with decreases in appetite (*r* = 0·11; *p* = 0·002), and decreases in regular protein intake (*r* = 0·07; *p* = 0·04). Non-significant associations included increases in appetite and consumption of sweets or unhealthy foods.

##### Eye health

Three studies^15^^,^^98^^,^^111^ reported six independent outcomes between screen time and eye health, with 83% (5/6) being statistically significant. Several types of screen time were significantly associated with myopic symptoms, including overall screen time (OR=1·26 95%CI 1·21–1·31), computer time (with TV time as the reference; OR=1·81 95%CI 1·05–3·12), smartphone use (with TV time as the reference; OR=2·02 95%CI 1·19–3·43), with multiple devices (with TV time as the reference) being non-significant.^15^ The progression of myopic symptoms was also associated with e-learning screen use (OR=1·07 95%CI 1·06–1·09).^111^ Furthermore, significant associations were found between digital device usage and digital eye strain (OR=3·6 95%CI 1·7–7·6).

##### Mental health

Eleven studies,^11^^,^^72^^,^^74^^,^^79^^,^^83^^,^^86^^,^^91^^,^ ^103^^,^^105^^,^^106^^,^^109^ with 86 independent outcomes examined screen time and aspects of mental health, with 29% (25/86) being significant.

Nine outcomes examined anxiety and screen time in children, of which 67% (6/9) were significant. Leisure time screen time was significantly associated with state anxiety in children of both healthy weight (*r* = 0·28; *p*=<0·05) and children who were overweight or obese (*r* = 0·20; *p*=<0·001).^11^ Playing online games (*r* = 0·11; *p*=<0·05), internet browsing (*r* = 0·21; *p*=<0·01), TV use (*r* = 0·16; *p*=<0·01), and social media use (*r* = 0·23; *p*=<0·01) were all also significantly associated with anxiety.^103^ Increases in overall screen time, however, was not significantly associated with anxiety.^86^

All four outcomes examining depression and screen time in children yielded significant associations, including between depression and: playing online video games (*r* = 0·12; *p*=<0·05); internet browsing (*r* = 0·21; *p*=<0·01); TV use (*r* = 0·16; *p*=<0·01); and social media use (*r* = 0·23; *p*=<0·01).^103^ Conversely, no significant associations were found between sadness and screen time in any of the seven independent outcomes.^72^^,^^86^

A total of 23 outcomes examined associations between screen time and behavioural factors, with 53% (9/17) of negative behavioural factors being statistically significant. Regarding overall screen time, significant associations were found in the following outcomes: aggression (β= 0·12; 95% CI 0·04 - 0·19); irritability (β= 0·12; 95% CI 0·06 - 0·19); frustration (β= 0·13; 95% CI 0·06 - 0·19); and frequency of temper tantrums (β= 0·10; 95% CI 0·03 - 0·17). Conversely, being afraid and being restless were not significant.^72^ Increasing total screen time was not associated with anger in any outcome,^86^ but increases in screen time were associated with fear (*p*=<0.01).^86^ Online study time was reported to be associated with mood disturbances (*r* = 0·43; *p*=<0·05), whereas leisure screen time was not significant.^109^ Tablet and smartphone time were both significantly associated with overall behavioural problems (*r* = 0·22; *p*=<0·05 and *r* = 0·17; *p*=<0·05 respectively), whereas TV time was not.^91^ Regarding other behavioural factors, child sensitivity, calmness, and affection were not associated with overall screen time.^72^^,^^86^

Seven outcomes examined associations between screen time and stress, with 50% (4/8) being significant. One study found a significant association between overall screen time and stress in a multiple mediation analysis (effect size=0·18; *p* = 0·05).^83^ Gaming was reported to be associated with ‘COVID related worries’ (OR=1·6 95%CI 1·1- 2·3), whereas TV time and social media use were not.^105^ Social media use was reported to be associated with ‘COVID related stress’ (OR=2·1; *p*=<0·001), whereas TV use and gaming were not.^103^ Increasing smartphone use was also reported to be associated with psychological distress (*r* = 0·2; *p*=<0·01).^79^

Regarding other aspects of mental health, studies reported increased odds of ‘at least two mental health problems from frequent sadness, irritability, and/or sleep problems’ (OR=2·51 *p*=<0·001).^106^ Negative affect was associated with leisure screen time in children with a healthy weight (*r* = 0·38; *p*=<0·05), but not in children who were overweight or obese.^11^ Positive affect was not associated with screen time in children of all weight categories.^11^ Other studies reported children's emotional and psychological well- being negatively associated with internet browsing (emotional *r*=−0·16; *p*=<0·01; psychological *r*=−0·13; *p*=<0·05) and social media use (emotional *r*=−0·12; *p*=<0·05; psychological *r*=−0·10; *p*=<0·05), but not with playing online games or TV use.^103^ The same study reported that children's social wellbeing was negatively associated (a protective effect) with internet browsing (*r*=−0·11; *p*=<0·05), but not with social media use, playing online games or TV use.^103^ The same study also reported no associations between children's resilience and internet browsing, social media use, TV use or playing online games.^103^

##### Physical activity

Regarding physical activity, four studies^11^^,^^74^^,^^76^^,^^90^ yielded 10 independent outcomes, with 70% (7/10) of outcomes being statistically significant. Overall screen time was negatively associated with physical activity (β= −0·18; 95%CI −0·25; −0·11) in one study,^90^ with another study (defining screen time as ‘the daily use of digital screens’) yielded non-significant results.^76^ Leisure screen time was positively associated with sedentary time in both healthy (*r* = 0·41; *p*=<0·05) and overweight/obese (*r* = 0·71; *p*=<0·05) children, however moderate/vigorous physical activity was not significant in both healthy and overweight/obese children.^11^ Other studies reported significant negative associations between overall physical activity and leisure (*r*= −0·16; *p*=<0·01), and education (*r*= −0·21; *p*=<0·01) screen time.^74^

##### Parental correlates

Five studies^74^^,^^83^^,^^95^^,^^96^^,^^109^ with 25 independent outcomes examined children's screen time and parental correlates, with 36% (9/25) being significant.

Overall screen time (as reported by the child) was negatively associated with parental screen time rules (β= −3·20; 95%CI −5·30; −2·19) and positively associated with the pandemic's impact on resources (β= 2·06; 95%CI 0·57 - 3·54), however was not significantly associated with: maternal stress; difficult balancing homelife; job/income loss; or difficulty obtaining childcare.^95^ Overall child screen time (as reported by the mother) in the same study yielded conflicting results, being negatively associated with parental awareness of social media (β=−3·37; 95%CI −4·20; −2·54), parental screen time rules (β =−3·81; 95%CI −5·43; −2·19), and positively associated with maternal stress (β =0·21; 95%CI 0·12 - 0·30). The pandemic's ‘impact on resources’; difficult balancing homelife; job/income loss; and difficulty obtaining childcare were non-significant.^95^ Overall screen time was also found to be significantly associated with parental screen use (*X*^2^=0·17; *p*=<0·05), but not parental employment status.^83^

Parental anxiety was significantly associated only with child video gaming (OR=1·78 95%CI 1·02–3·11),^96^ and not with overall screen time^83^^,^^96^ or TV use.^96^ Conflicts with parents were also associated with both online (β=0·02; *p*=<0.05) and leisure based (β=0·06; *p*=<0.01) screen time.^109^ Parental stress was not associated with overall screentime,^83^ screen time for edication,^74^ or screen time for leisure.^74^

##### Weight gain/BMI

Two studies^88^^,^^99^ yielding four independent outcomes reported associations between physiology and screen time, with no significant findings reported between screen time and changes in BMI^99^ and several categorical weight variables.^88^

##### Sleep

Regarding sleep, five studies^76^^,^^90^, ^91^, ^92^^,^^107^ with seven independent outcomes were included, of which 86% (6/7) were significant.

Sleep duration yielded conflicting results, with one study reporting negative correlations between overall screen time and sleep duration (*r*=−0·40; *p*=<0·01),^76^ and another reporting positive associations (β = 0·003; 95% CI 0·001 - 0·005).^90^ Non-academic screen time was negatively associated with sleep duration (*r*=−0·41; *p*=<0·01).^92^

Overall screen time was also associated with increased odds of sleep disorders (OR=3·80 95%CI 1·09–13·1),^107^ and tablet (*r* = 0·17; *p*=<0·05) and smartphone time (*r* = 0·30; *p*=<0·001) were both associated with ‘sleep problems’ (TV time was not significantly associated with sleep problems).^91^

##### Problematic screen time behaviours

Two studies^79^^,^^81^ yielding four independent outcomes were found reporting associations between screen time and problematic screen time behaviours, all of which were statically significant. Gaming time was reported to be significantly associated with gaming disorder symptoms (*r* = 0·43; *p*=<0·001),^81^ and smartphone use was reported to be significantly associated with problematic smartphone use (*r* = 0·35; *p*=<0·01), problematic social media use (*r* = 0·29; *p*=<0·01), and problematic gaming (*r* = 0·25; *p*=<0·01).^79^

## Discussion

This systematic review and meta-analysis, including 89 studies, examined the pooled reported changes in screen time from before the COVID-19 pandemic, and narratively examined correlates associated with screen time during the COVID-19 pandemic.

The results from the meta-analysis showed that all groups significantly increased both their total and leisure screen time. Children of primary age had the largest increase in both total and leisure screen time, followed by adults, with adolescents and young children yielding the smallest increase. Furthermore, 51% of adults and 67% of children reported increases in total screen time, and 52% of adults and 60% of children reported increases in leisure screen time. These results are in line with research showing increases in sedentary behaviours during the COVID-19 pandemic,^4^ although this study is the first to examine screen time independently. Although the increases in total screen time could be partially caused by increasing time in front of a screen for work or academic purposes (such as increases in online meetings and education), the increases in non-academic screen time in children are concerning. Indeed, it has been reported in pre-COVID-19 reviews that screen time is associated with several unfavourable outcomes, such as increased BMI, increased maternal depression, lower cognitive stimulation at home, decreased quality of life, lower self-esteem, and anxiety.^112^^,^^113^ A recent systematic review and meta-analysis has also concluded that smart device exposure may be associated with increased risk of myopia in children, indicating that increases in screen time during COVID may also lead to increased prevalence of myopia.^16^ Furthermore, a recent longitudinal study has reported that screen time at the age of 4 is negatively associated with mathematic and literacy grades at the age of 8,^114^ suggesting that screen time at a younger age could affect future academic achievement. Although the absolute screen time adults and children should adhere to are under debate and not universally agreed upon, there is a growing consensus that leisure screen time should be minimised in favour of physically active pursuits.^115^ Moreover, the UK Chief Medical Officer recommends that parents of children (of all ages) proactively consider if a child's screen time if affecting sleep, physical activity, and snacking.^116^

Several negative food behaviours were associated with increases in screen time in both adults and children. These included associations between increased screen time and ‘unhealthy diet changes’, and associations between increased computer, television, and cell phone use and increases in sweet food consumption. Further, increases in computer time for self-directed learning was found to be positively associated with eating related self-regulation while increases in TV time were found to be negatively correlated, and other types of screen time, including computer gaming and overall screen time, were not significantly associated. Importantly, there were more significant associations between increases in screen time in children than in adults, suggesting that appropriate interventions may benefit children more. This broadly concurs with pre-pandemic reviews showing sedentary behaviours (including screen time) are associated with less healthy diets, including lower fruit and vegetable consumption, higher energy dense drinks, fast foods and higher total energy intake in both adults and children.^117^^,^^118^ As the pandemic has resulted in increases in screen time, it is recommended that public health guidance on reduced screen time and healthy dietary behaviours during it be promoted, with a focus on targeting parents and children.

Alcohol use during the pandemic was shown to be negatively associated with increased computer time, however increased television use was associated with an increased desire to drink alcohol (but not with increased consumption). Although other reviews have reported increases in alcohol consumption during the pandemic,^119^ results from this study suggest that these increases are not associated with screen time. Longitudinal study is warranted to examine these behaviours. Furthermore, all correlates related to smoking (smoking and desire to smoke) were not significantly associated with any type of screen time use, indicating that COVID-19 screen time did not affect smoking habits.

This review found that several eye related correlates were associated with screen time. In adults, increases in screen time was consistently associated with dry eye syndrome, which broadly agrees with previous literature in both a COVID-19 and non-COVID-19 context.^120^^,^^121^ It was also found that more than 6 h of screen time/day was associated with several symptoms of digital eye strain, including tearing, eye strain, dryness, heavy eyelids, red eyes, eye itchiness, burning sensation in the eye, sensitivity of bright light, difficulty focussing, eye pain, the feeling of a foreign body in the eye, and excessive blinking. These results concur with pre-pandemic reviews that have found associations between dry eye syndrome and screen time.^120^ It has previously been reported that the possible mechanisms for this could be reduced blink rates, meibomian gland dysfunction, and corneal phototoxicity, most likely to be multifactorial,^120^and further study is warranted, especially longitudinal study to establish temporal relationships.

In children, increased screen time was significantly associated with myopia, with almost all associations being significant. Indeed, every stratified type of screen time significantly correlated with increases in myopia across multiple studies, with the exception of ‘multiple devices’. This is in agreement with a recent meta-analysis that reported associations between screen time and myopia,^16^ however other reviews have reported mixed results.^122^ Although the results from this review cannot determine temporal relationships due to the cross-sectional design of included studies, these results concur with longitudinal studies that have found that increased screen time may be a casual factor of myopia in children.^123^ It is recommended that children minimise screen time (particularly using screens where the child is very close to the screen, such as tablets and phones) use to potentially prevent dry eye and increased risk of myopia. As higher odds of myopia were found in increased smartphone devices, it is also recommended that any e-learning be conducted on a larger screen further away from the eyes, and not on a smartphone device (or similar), to negate these risks.

In adults, studies that measured overall screen time subjectively mostly found significant associations, however the two studies that objectively measured screen time found no significant associations. Regarding different types of screen time, one study found that TV time was associated with COVID-19 related anxiety, with other studies finding no association between TV time and overall anxiety. Due to these conflicting results, it is difficult to come to conclusions regarding screen time and anxiety. This is broadly in agreement with other systematic reviews examining sedentary behaviour (including screen time) and anxiety in adults.^124^ We agree with Teychenne, Costigan and Parker^124^ that large longitudinal studies are needed to comprehensively examine this possible association.

In children, there was a general consensus across included studies that overall screen time was not associated with anxiety, however this was not the case regarding stratified screen time. Indeed, studies reported associations between anxiety and leisure screen time, online gaming, internet browsing, TV and social media use, with no stratified type of screen time yielding non-significant results. This is in agreement with other large longitudinal studies that have found associations between screen time and anxiety in adolecents,^125^^,^^126^ however more research is needed in children of younger ages.

In adults, studies reported conflicting results, with different studies reporting significant associations between overall screen time and depression in both directions (e.g. one study showed a protective effect), with other studies reporting no association. The only stratified type of screen time was TV, which was found to be significantly associated. It is likely that these conflicting results are because of heterogeneity in populations, measurement tools, and statistical methodology. Although previous systematic reviews have reported positive associations between screen time and depression in adults, in one review the significance of results changed when stratifying according to gender (only females yielded a significant association).^127^ Furthermore, other studies have found that only moderate to severe depression is associated with screen time.^128^ It is therefore difficult to conclude whether depression is associated with screen time during the COVID-19 pandemic – further studies with heterogeneous measurement tools would be highly beneficial.

In contrast to adults, the studies that examined screen time and depression in children all found significant associations, suggesting a link between screen time and depression in children with a higher level of certainty. This is in agreement with previous literature that has concluded that screen time is associated with depression in children, with a significant (non-linear) dose response relationship.^129^ Although the direction of association is difficult to ascertain, it is recommended that parents monitor screen time usage in children to prevent or identify possible depressive symptoms.

Studies that examined mood changes yielded non-significant results in adults but showed several mood changes that were significantly associated with screen time in children. These significant associations, across several studies included increased aggression, irritability, frustration, temper tantrums, and mood disturbances. When stratified according to type of screen time, personal devices, such as mobile phones and tablets were associated with behavioural problems in children, whereas TV time was not. This concurs with a pre-pandemic umbrella review that found weak evidence for associations between poor mental health outcomes and screen time in children and adolescents.^130^ As with adults, it is currently unknown as to the mechanisms that drive these associations, and whether they are chronic or acute. For example, a review examining longitudinal studies found no longitudinal associations between increased screen time as a child and most long-term mental health conditions.^131^

In adults, most studies reported significant (direct whilst indirect were not significant) associations between several types of screen time (including overall screen time) and loneliness, however in one study this was only found in people without concurrent depression. This is in broad agreement with other pre-COVID studies that have found associations between screen time and loneliness, with some studies reporting that decreases in screen time (in particular social media use) can decrease loneliness.^132^

In children, several stress-related correlates were significantly associated with screen time, while others were not. Although a significant association was found between overall screen time and stress, this was found in only one study. Gaming related screen time was found to be associated with COVID-related worries, but not COVID-related stress. Social media use, on the other hand, was found to be associated with COVID-related stress, but not with COVID-related worries. Smartphone use was associated with psychological distress in one study. Lastly, TV time was not significantly associated with any form of stress in any study. Overall, the evidence is mixed, however previous studies have reported associations between screen time and stress in children.^133^ This could be because there were no more than one outcome examining the same correlate (except from TV use), and further research is warranted.

Other significant correlates of mental health in adults and children included several types of screen time being associated with general mental health and wellbeing, which concurs with several previous studies that have concluded similar results.^127^^,^^133^ Although when stratified according to type of screen time and type of mental health correlate, it is clear that several of the included studies agree with pre-COVID studies that increases in screen time are linked to negative mental health outcomes.^127^^,^^133^^,^^134^ It is therefore recommended that screen time be reduced wherever possible (for example, leisure time screen time) to negate these negative outcomes.

Overall screen time was consistently associated with decreases in physical activity in adults, however conflicting results were found regarding TV time. Furthermore, gaming and social media use were both found to correlate with physical activity, however smartphone use and PC/computer/tablet use were not. Weight gain was associated with time spent lying in front of a TV screen, yet in a different study overall BMI was not associated with overall screen time. Few previous studies have reported associations between screen time and physical activity levels, however studies have reported negative outcomes in adults with high screen time and low physical activity levels, including health related quality of life.^135^ Although screen time is generally classified as a sedentary behaviour, there are types of screen time that promote physical activity, including exergaming (a type physical activity that is technology-driven, and often includes an element of screen time), a type which has not been explicitly identified in this review. Indeed, exergaming has been shown to reduce anxiety levels and increase physical activity levels,^136^ and has been postulated as a potential source of physical activity during the COVID-19 pandemic, particularly in times of quarantine.^137^^,^^138^ Primary studies regarding the efficacy and accessibility of exergaming as an alternative to sedentary based screen time behaviours during the COVID-19 pandemic are warranted.

Significant associations between screen time and physical activity in children were conflicting, with some studies reporting negative associations between physical activity and overall, leisure, and education screen time, whereas other studies reported null results. The conflicting results could be due to several factors, including reporting biases and statistical methodology. Regarding sedentary behaviour, screen time was consistently associated in children, however changes in BMI and weight gain were not associated with screen time in any study. This concurs with much of the literature that has found similar associations, predominantly because screen time is usually conducted while in a sedentary position.^130^ It also agrees with previous reviews that have shown large increases in sedentary behaviour during the pandemic.^4^ Because several previous studies have found significant negative associations between screen time and physical activity in children of all ages, increases in physical activity and corresponding decreases in screen time being recommended.^139^, ^140^, ^141^

In adults, studies agreed that screen time was not associated with sleep or fatigue correlates, including sleep time. This is in conflict with pre-COVID studies that have found association between screen time and sleep time in adults.^142^ This conflict may be because people have been reported to experience sleep disturbances as a result of the COVID-19 pandemic.^143^

Conversely, in children, several studies agreed that increases in screen time were associated with sleep problems, including sleep duration and sleep disorders. The only exception to this appears to be TV time, which was not found to be associated with sleep problems. This is in broad agreement with much of the literature that reports disturbed sleep patterns with increases in screentime.^17^^,^^18^^,^^130^ All of these correlations, however, did not adjust for other known correlates of sleep disturbances, such as anxiety and depression.^144^ Further examination of potential mediating factors is warranted.

Several parental anxiety and parental stress correlates were associated with different types of screen time. Furthermore, conflicts with parents were significantly associated with increases in screen time, although these results should be treated with caution as several correlates were only measured in one study. This concurs with pre-pandemic reviews that have reported that parental stress is associated with child screentime.^112^^,^^145^ One possible reason for this finding is that COVID-related parental COVID-related stress is having a direct impact on child screen time – further longitudinal studies are required to establish temporal relationships.

There was a consensus across studies that screen time was associated with negative screen related behaviours, including problematic gaming and social media use. This is in agreement with the literature, and is a concerning finding because previous studies have reported that problematic screen use is associated with several negative mental health outcomes.^12^ Furthermore, it has been reported in a recent systematic review and meta-analysis that problematic smartphone behaviours are increasing globally,^146^ however the majority of included studies (as well as previous literature concerning screen time) did not include problematic usage in their studies.^12^ It is recommended, therefore, that both screen time and problematic screen time behaviours be monitored in children closely, and sedentary based leisure time screen time be reduced in favour of other activities that have been shown to have positive effects on mental and physical health, including physical activity and exergaming (especially if people are subject to further periods of restrictions).

Although this is the first review examining screen time changes during the COVID-19 pandemic in adults and children, the results of this review should be taken within its limitations. Firstly, there was high heterogeneity in the meta-analysis, which we could not fully explain. It is likely that differing methods of measuring screen time, and different populations, contributed to this. Furthermore, there was a large range in the quality of studies included, which may have added to the heterogeneity. Secondly, although several significant effect sizes were found spanning a wide variety of unfavourable outcomes, several non-significant findings were also found, possibly due to the heterogeneous nature of the included populations and the measures used. Thirdly, the methods of measuring screen time were highly heterogeneous, with some studies using self-report, others using parental report, and others using objective reports – future research should use either validated measures of screen time and/or problematic screen time use, or objective measures of screen time wherever possible. Lastly, although there were several correlates that concurred between studies, other significant correlates were based on one effect size from one study, and therefore the results of these should be treated with caution.

In conclusion, this review has found evidence that both overall and leisure screen time increased during the COVID-19 pandemic, with children of primary school age yielding the highest increases. Furthermore, several unfavourable correlates have been reported to be associated with increases in screen time in both adults and children, including several mental health correlates. In addition to the well-reported benefits of physical activity, it is recommended that leisure screen time should be reduced in favour of non-sedentary activities, including physical activity, especially in children. If, however, physical activity is difficult (for example, in periods of restrictions limiting the ability to go outdoors), screen related physical activity such as exergaming may yield favourable outcomes.

### Data sharing statement

All data used in this research was gathered from already existing research. No original data was used for this study. Data extraction tables and figures are available from MT.

## Contributors

Mike Trott: conceptualisation; literature search; figures; study design; data collection; data analysis; data interpretation; writing, Robin Driscoll: literature search; data collection; data analysis; writing, Enrico Iraldo: literature search; data collection; writing, Shahina Pardhan: conceptualisation; study design; writing; supervision.

## Declaration of interests

None to report.

## Appendix Supplementary materials

Image, application 1
