# Supplementary material for: Changes and correlates of screen time in adults and children during the COVID-19 pandemic: A systematic review and meta-analysis
Source: eClinicalMedicine. 2022 May 21;48:101452. doi: 10.1016/j.eclinm.2022.101452 (PMC9122783; doi:10.1016/j.eclinm.2022.101452)
Supplement: Supplementary file 1 [file mmc1.docx]

**Supplementary Table 1: Full NOS scoring**

| **Author(s)** | **Selection** | | | | **Comparability** | **Outcome** | | **Total 'stars'** |
| --- | --- | --- | --- | --- | --- | --- | --- | --- |
|  | **Representativeness of the sample** | **Sample size** | **Non-respondents** | **Ascertainment of the exposure** | **The subjects in different outcome groups are comparable, based on the study design or analysis. Confounding factors are controlled.** | **Assessment of the outcome** | **Statistical test** |  |
| Abdulsalam et al. | 0 | 0 | 0 | 1 | 1 | 1 | 1 | 4 |
| Abid et al. | 1 | 1 | 0 | 2 | 1 | 2 | 1 | 8 |
| Aguilar-Farias et al. | 1 | 0 | 0 | 2 | 1 | 2 | 1 | 7 |
| Agurto et al. | 1 | 0 | 0 | 2 | 1 | 2 | 1 | 7 |
| Alomari et al. | 1 | 0 | 0 | 2 | 1 | 2 | 1 | 7 |
| Alves et al. | 1 | 0 | 0 | 2 | 1 | 2 | 1 | 7 |
| Balsam | 1 | 1 | 0 | 2 | 1 | 1 | 1 | 7 |
| Beck et al. | 1 | 0 | 0 | 2 | 1 | 1 | 0 | 5 |
| Bird et al. | 1 | 0 | 0 | 2 | 1 | 2 | 1 | 7 |
| Branquinho et al. | 0 | 0 | 0 | 1 | 1 | 1 | 1 | 4 |
| Breidokiene et al. | 1 | 0 | 0 | 1 | 2 | 1 | 1 | 6 |
| Brzek et al. | 1 | 0 | 1 | 1 | 1 | 1 | 1 | 6 |
| Cachon-Zagalaz et al. | 1 | 0 | 0 | 1 | 1 | 2 | 1 | 6 |
| Cahal et al. | 1 | 0 | 0 | 2 | 1 | 2 | 1 | 7 |
| Chambonniere et al. | 1 | 0 | 0 | 2 | 1 | 2 | 1 | 7 |
| Cheikh Ismail et al. | 1 | 0 | 0 | 2 | 1 | 2 | 1 | 7 |
| Chen et al. | 1 | 1 | 0 | 2 | 1 | 1 | 1 | 7 |
| Conroy et al. | 1 | 0 | 0 | 1 | 1 | 2 | 1 | 6 |
| Constandt et al. | 1 | 1 | 0 | 2 | 1 | 1 | 1 | 7 |
| Coyne et al. | 1 | 0 | 1 | 1 | 0 | 1 | 1 | 5 |
| de Sa et al. | 1 | 0 | 0 | 1 | 1 | 2 | 1 | 6 |
| Donati et al. | 1 | 0 | 0 | 2 | 2 | 1 | 1 | 7 |
| Dragun et al. | 1 | 0 | 0 | 1 | 1 | 2 | 1 | 6 |
| Dubuc et al. | 1 | 0 | 0 | 1 | 1 | 1 | 1 | 5 |
| Farah et al. | 1 | 0 | 0 | 2 | 1 | 2 | 1 | 7 |
| Fillon et al. | 1 | 0 | 0 | 1 | 1 | 1 | 0 | 4 |
| Fraser et al. | 1 | 0 | 0 | 2 | 2 | 1 | 1 | 7 |
| Ganne et al. | 0 | 0 | 1 | 2 | 1 | 1 | 1 | 6 |
| Garcia et al. | 0 | 0 | 0 | 2 | 1 | 1 | 1 | 5 |
| Genin et al. | 1 | 0 | 0 | 2 | 1 | 2 | 1 | 7 |
| Giannini et al. | 1 | 1 | 0 | 1 | 1 | 1 | 1 | 6 |
| Gornika et al. | 1 | 0 | 1 | 1 | 1 | 1 | 1 | 6 |
| Guo et al. | 1 | 1 | 1 | 1 | 2 | 1 | 1 | 8 |
| Hadianfard et al. | 1 | 0 | 0 | 2 | 1 | 2 | 1 | 7 |
| Hashem et al. | 1 | 1 | 0 | 1 | 2 | 1 | 1 | 7 |
| Helbach and Stahlmann | 1 | 0 | 0 | 2 | 1 | 1 | 1 | 6 |
| Hodes et al. | 1 | 0 | 0 | 2 | 1 | 2 | 1 | 7 |
| Hu et al. | 1 | 0 | 1 | 2 | 1 | 1 | 1 | 7 |
| Hyunshik et al. | 1 | 0 | 1 | 1 | 2 | 1 | 1 | 7 |
| Jáuregui et al. | 1 | 0 | 0 | 2 | 1 | 2 | 1 | 7 |
| Jia et al. | 1 | 0 | 0 | 2 | 1 | 1 | 1 | 6 |
| Kim et al. | 1 | 1 | 0 | 2 | 1 | 2 | 1 | 8 |
| Koohsari et al. | 1 | 0 | 1 | 1 | 1 | 1 | 1 | 6 |
| Kowalsky et al. | 1 | 0 | 0 | 2 | 1 | 2 | 1 | 7 |
| Lawrence et al. | 1 | 1 | 0 | 1 | 1 | 1 | 1 | 6 |
| Le et al. | 1 | 0 | 0 | 1 | 1 | 2 | 1 | 6 |
| Lim et al. | 1 | 0 | 0 | 1 | 1 | 2 | 1 | 6 |
| Liu et al.a | 1 | 0 | 1 | 1 | 2 | 1 | 1 | 7 |
| Lui et al. b | 1 | 0 | 1 | 1 | 1 | 1 | 1 | 6 |
| Lopez-Gil et al. | 1 | 0 | 0 | 1 | 1 | 2 | 1 | 6 |
| Ma et al. | 0 | 0 | 0 | 2 | 1 | 1 | 1 | 5 |
| Majumdar et al. | 1 | 0 | 0 | 2 | 1 | 2 | 1 | 7 |
| McArthur et al. | 1 | 0 | 0 | 2 | 1 | 2 | 1 | 7 |
| McCormack et al. | 1 | 0 | 0 | 2 | 1 | 2 | 1 | 7 |
| Meyer et al. | 1 | 0 | 0 | 1 | 1 | 2 | 1 | 6 |
| Mitra et al. | 1 | 0 | 0 | 1 | 1 | 2 | 1 | 6 |
| Mohan et al. | 1 | 0 | 0 | 1 | 1 | 2 | 1 | 6 |
| Mon-Lopez et al. | 0 | 0 | 1 | 1 | 1 | 1 | 1 | 5 |
| Nassar et al. | 0 | 0 | 0 | 2 | 1 | 2 | 1 | 6 |
| Nathan et al. | 1 | 0 | 0 | 1 | 1 | 1 | 1 | 5 |
| Oswald et al. | 1 | 0 | 0 | 2 | 1 | 2 | 1 | 7 |
| Pavithra and Sundar | 1 | 1 | 0 | 1 | 0 | 2 | 1 | 6 |
| Peddie et al. | 1 | 1 | 1 | 1 | 1 | 1 | 1 | 7 |
| Robbins et al. | 1 | 0 | 0 | 2 | 2 | 1 | 1 | 7 |
| Rodriguez-Larrad et al. | 1 | 0 | 0 | 2 | 1 | 2 | 1 | 7 |
| Sallie et al. | 1 | 0 | 1 | 2 | 2 | 1 | 1 | 8 |
| Sanudo et al. | 1 | 1 | 0 | 1 | 1 | 2 | 1 | 7 |
| Saxena et al. | 0 | 0 | 0 | 1 | 1 | 2 | 0 | 4 |
| Schmidt et al. | 0 | 0 | 0 | 2 | 1 | 2 | 1 | 6 |
| Sewall et al. | 0 | 0 | 0 | 1 | 1 | 2 | 1 | 5 |
| Sikorska et al. | 1 | 0 | 0 | 2 | 2 | 1 | 1 | 7 |
| Siste et al. | 1 | 0 | 0 | 2 | 2 | 1 | 1 | 7 |
| Spence et al. | 1 | 0 | 0 | 2 | 1 | 2 | 1 | 7 |
| Stieger et al. | 1 | 0 | 0 | 1 | 1 | 2 | 1 | 6 |
| Stokes et al. | 1 | 0 | 0 | 1 | 2 | 1 | 1 | 6 |
| Suka et al. | 1 | 0 | 0 | 2 | 1 | 2 | 1 | 7 |
| Szwarcwald et al. | 1 | 0 | 0 | 1 | 1 | 2 | 1 | 6 |
| Tan et al. | 1 | 1 | 0 | 2 | 2 | 1 | 1 | 8 |
| Tebar et al. | 1 | 0 | 0 | 1 | 1 | 2 | 0 | 5 |
| Werneck et al. | 1 | 0 | 0 | 1 | 1 | 2 | 1 | 6 |
| Windiani et al. | 1 | 0 | 0 | 2 | 1 | 2 | 1 | 7 |
| Woodruff et al. | 1 | 0 | 0 | 2 | 1 | 2 | 1 | 7 |
| Wunsch et al. | 1 | 0 | 0 | 1 | 1 | 2 | 1 | 6 |
| Xiao et al. | 1 | 0 | 0 | 1 | 1 | 2 | 1 | 6 |
| Yang et al. | 1 | 0 | 0 | 2 | 1 | 2 | 1 | 7 |
| Zajacova et al. | 1 | 0 | 0 | 2 | 1 | 2 | 1 | 7 |
| Zarco-Alpeunte et al. | 0 | 0 | 1 | 2 | 1 | 1 | 1 | 6 |
| Zhang et al. | 1 | 0 | 0 | 1 | 1 | 2 | 1 | 6 |
| Zhou et al. | 1 | 0 | 0 | 1 | 1 | 2 | 1 | 6 |

**Supplementary Table 2: Reported associations between screen time and any outcome in adults**

| **Type of association** | **Author(s)** | **Type of screen time**  **(dependent variable)** | **Type of correlation**  **(independent variable)** | **Effect size type** | **Effect size**  **(95% CI)** | **p-value** | **Adjustments** |
| --- | --- | --- | --- | --- | --- | --- | --- |
| Diet and smoking | Gornika et al. | Increases in screen time | Pro-healthy diet changes | OR | 1·17  (0·94-1·44) | NS | BMI; GDP |
|  |  |  | Diet constant | OR | 0·68  (0·56-0·82) | <0·001 |  |
|  |  |  | Unhealthy diet changes | OR | 1·54  (1·21-1·96) | <0·001 |  |
|  | Tebar et al. | Increased television use | Alcohol consumption | OR | 0·97  (0·78-1·21) | NS | Age, sex, educational level, feeling of stress, feeling of anxiety, feeling of depression, use of screen device for physical activity, and total screen time per day |
|  |  | Increased cell phone use | Alcohol consumption | OR | 1·08  (0·79-1·48) | NS |  |
|  |  | Increased computer time | Alcohol consumption | OR | 0·68  (0·53-0·86) | <0·05 |  |
|  |  | Increased television use | Increased desire to drink alcohol | OR | 1·46  (1·12-1·89) | <0·05 |  |
|  |  | Increased cell phone use | Increased desire to drink alcohol | OR | 1·24  (0·84-1·82) | NS |  |
|  |  | Increased computer time | Increased desire to drink alcohol | OR | 0·87  (0·66-1·14) | NS |  |
|  |  | Increased television use | Sweetened foods consumption | OR | 1·02  (0·83-1·27) | NS |  |
|  |  | Increased cell phone use | Sweetened foods consumption | OR | 1·01  (0·73-1·38) | NS |  |
|  |  | Increased computer time | Sweetened foods consumption | OR | 0·78  (0·62-0·98) | <0·05 |  |
|  |  | Increased television use | Increased sweetened foods consumption | OR | 1·53  (1·18-1·99) | <0·05 |  |
|  |  | Increased cell phone use | Increased sweetened foods consumption | OR | 1·78  (1·18-2·67) | <0·05 |  |
|  |  | Increased computer time | Increased sweetened foods consumption | OR | 1·16  (0·88-1·53) | NS |  |
|  |  | Increased television use | Increased smoking | OR | 0·87  (0·55-1·38) | NS |  |
|  |  | Increased cell phone use | Increased smoking | OR | 0·78  (0·42-1·43) | NS |  |
|  |  | Increased computer time | Increased smoking | OR | 0·71  (0·44-1·14) | NS |  |
|  |  | Increased television use | Increased desire to smoke | OR | 0·58  (0·31-1·09) | NS |  |
|  |  | Increased cell phone use | Increased desire to smoke | OR | 0·58  (0·24-1·4) | NS |  |
|  |  | Increased computer time | Increased desire to smoke | OR | 0·6  (0·30-1·18) | NS |  |
|  | Tan et al | Television viewing | Eating self-regulatory score | Pearson's r | -0.24 | 0.01 | NA |
|  |  | Computer games | Eating self-regulatory score | Pearson's r | -0.06 | NS |  |
|  |  | Console games | Eating self-regulatory score | Pearson's r | -0.15 | 0.04 |  |
|  |  | Internet (non-study) | Eating self-regulatory score | Pearson's r | -0.09 | NS |  |
|  |  | Internet (online lectures) | Eating self-regulatory score | Pearson's r | -0.03 | NS |  |
|  |  | Internet (self-directed learning) | Eating self-regulatory score | Pearson's r | 0.17 | 0.02 |  |
|  |  | Total screen time | Eating self-regulatory score | Pearson's r | -0.11 | NS |  |
| Eye health | Pavithra and Sundar | Increased screen time | Dry eye syndrome | OR | 66·7  (20·4-218·3) | <0·001 | None |
|  | Balsam | Less than 6 hours of screen time/day with more than 6 hours of screen time/day as the reference group. | Dry eye syndrome | OR | 0·51  (0·39-0·67) | <0·001 | NR |
|  |  |  | Tearing | OR | 0·72  (0·54-0·96) | 0·03 | NR |
|  |  |  | Eye strain | OR | 0·51  (0·41-0·64) | <0·001 | NR |
|  |  |  | Dryness | OR | 0·62  (0·49-0·79) | <0·001 | NR |
|  |  |  | Heavy eyelids | OR | 0·68  (0·51-0·91) | 0·01 | NR |
|  |  |  | Eye redness | OR | 0·60  (0·44-0·81) | <0·001 | NR |
|  |  |  | Blurred vision | OR | 1·01  (0·79-1·28) | NS | NR |
|  |  |  | Diplopia | OR | 0·88  (0·62-1·26) | NS | NR |
|  |  |  | Eye itchiness | OR | 0·53  (0·40-0·69) | <0·001 | NR |
|  |  |  | Burning sensation in the eye | OR | 0·59  (0·45-0·76) | <0·001 | NR |
|  |  |  | Sensitivity of bright light | OR | 0·58  (0·43-0·79) | <0·001 | NR |
|  |  |  | Difficulty focusing | OR | 0·70  (0·55-0·90) | <0·005 | NR |
|  |  |  | Eye pain | OR | 0·56  (0·41-0·75) | <0·001 | NR |
|  |  |  | Foreign body sensation in the eye | OR | 0·69  (0·49-0·98) | 0·04 | NR |
|  |  |  | Excessive blinking | OR | 0·68  (0·52-0·87) | <0·003 | NR |
|  | Ganne et al. | Total screen time | Dry eye syndrome | Chi square | 39.2 (df=4) | <0.001 | NA |
| Mental health | Balsam | Less than 6 hours of screen time/day with more than 6 hours of screen time/day as the reference group. | Headache | OR | 0·55  (0·44-0·70) | <0·001 | NR |
|  | Koohsari et al. | Television viewing | Concentration | Unstandardised regression coefficient | 0·11  (-0·04-0·26) | NS | Age, sex, marital status, highest education, gross annual household income, and baseline fatigue |
|  |  | PC use sitting time | Concentration | Unstandardised regression coefficient | -0·11  (-0·26 – 0·04) | NS |  |
|  | Zhang et al. | 3-4hr screen time  (≤2hr as the reference) | Anxiety  (dichotomous variable) | OR | 1·38  (0·93-2·04) | NS | Age, pre-pregnant BMI, gestational weeks, residence, occupation, psychologic situation during pandemic of COVID-19, timely prenatal examinations, cut off of health care products, household income during pandemic of COVID-19, rhythm of life during COVID-19, education, physical frequency, and sleep duration |
|  |  | 5-6hr screen time  (≤2hr as the reference) | Anxiety  (dichotomous variable) | OR | 1·76  (1·20-2·58) | <0·05 |  |
|  |  | 7-8hr screen time  (≤2hr as the reference) | Anxiety  (dichotomous variable) | OR | 1·98  (1·29-3·03) | <0·05 |  |
|  |  | ≥8hr screen time  (≤2hr as the reference) | Anxiety  (dichotomous variable) | OR | 2·22  (1·45-3·40) | <0·05 |  |
|  | Meyer et al. | Screen time | Depression | Unstandardised regression coefficient | 1·924 (SE=0·441) | <0·001 | Age, sex, race, smoking, relationship status, employment, chronic illnesses, and COVID-19 public health restrictions |
|  |  | Screen time | Anxiety | Unstandardised regression coefficient | 1·341 (SE=0·454) | 0·003 |  |
|  |  | Screen time | Loneliness | Unstandardised regression coefficient | 0·340 (SE=0·095) | <0·001 |  |
|  |  | Screen time | Stress | Unstandardised regression coefficient | 0·590 (SE=0·154) | <0·001 |  |
|  |  | Screen time | Social Network | Unstandardised regression coefficient | −0·069 (SE=0·145) | NS |  |
|  |  | Screen time | Positive mental health | Unstandardised regression coefficient | −0·920 (SE=0·239) | <0·001 |  |
|  | Koohsari et al. | PC use sitting time during workday | Concentration | Unstandardised regression coefficient | -0·13  (-0·28-0·03) | NS | Age, sex, marital status, highest education, gross annual household income, and baseline fatigue |
|  |  | PC use sitting time during workday | Motivation | Unstandardised regression coefficient | -0·02  (-0·16-0·13) | NS |  |
|  | Stieger et al. | Screen time | Wellbeing | ICC | -0·31  (-0·53; -0·01) | <0·01 | NR |
|  |  | Screen time * loneliness | Wellbeing | ICC | -0·01  (-0·16-18·0) | NS | NR |
|  | Hu et al. | Increased leisure time screen time | Subjective wellbeing | OR | 1·03  (0·80-1·34) | NS | Age, gender, marital status, residential location, education, and personal monthly income, plus self-rated physical health, perceived social support, and loneliness. |
|  | Oswald et al. | Increasing screen time | ‘Languishing’ vs ‘flourishing’ mental health | RR | 1·42  (0·83-2·37) | NS | Gender, studying (yes/no), SES, and other screen time experience variables |
|  |  | Increasing screen time | ‘Struggling’ vs ‘flourishing’ mental health | RR | 2·20  (1·32-3·65) | <0·05 |  |
|  |  | Increasing screen time | ‘Floundering’ vs ‘flourishing’ mental health | RR | 1·18  (0·65-2·17) | NS |  |
|  |  | Decreasing screen time | ‘Languishing’ vs ‘flourishing’ mental health | RR | 4·53  (0·95-21·72) | NS |  |
|  |  | Decreasing screen time | ‘Struggling’ vs ‘flourishing’ mental health | RR | 23·85  (5·44-104·4) | <0·05 |  |
|  |  | Decreasing screen time | ‘Floundering’ vs ‘flourishing’ mental health | RR | 3·27  (0·58-18·37) | NS |  |
|  | Sewall et al. | Screen time (objectively measured) | Depression | Unstandardised regression coefficient | -0·01  (-0·03-0·02) | NS | Demographic variables |
|  |  | Screen time (objectively measured) | Anxiety | Unstandardised regression coefficient | -0·02  (-0·04-0·01) | NS | Demographic variables |
|  |  | Screen time (objectively measured) | Suicidal ideation | Unstandardised regression coefficient | 0·01  (-0·01-0·02) | NS | Demographic variables |
|  | Werneck et al. | Television use ‘consistently low’ pre-COVID versus ‘become low’ post=-COVID [undefined in paper] | Loneliness in persons without depression | OR | 0·74  (0·42-1·3) | NS | Sex, age group, highest academic achievement, working status during the pandemic, skin color, alcohol use, tobacco smoking, diagnoses of COVID-19 on a close friend, co-worker or relative and adherence to the quarantine |
|  |  | Television use ‘consistently low’ pre COVID versus ‘become high’ post-COVID [undefined in paper] | Loneliness in persons without depression | OR | 1·59  (1·37-1·86) | <0·05 |  |
|  |  | Television use ‘consistently low’ pre COVID versus ‘consistently high’ post COVID [undefined in paper] | Loneliness in persons without depression | OR | 1·15  (0·90-1·47) | NS |  |
|  |  | Television use ‘consistently low’ pre-COVID versus ‘become low’ post=-COVID [undefined in paper] | Sadness in persons without depression | OR | 1·26  (0·69-2·30) | NS |  |
|  |  | Television use ‘consistently low’ pre COVID versus ‘become high’ post-COVID [undefined in paper] | Sadness in persons without depression | OR | 1·68  (1·44-1·96) | <0·05 |  |
|  |  | Television use ‘consistently low’ pre COVID versus ‘consistently high’ post COVID [undefined in paper] | Sadness in persons without depression | OR | 1·12  (0·86-1·45) | NS |  |
|  |  | Television use ‘consistently low’ pre-COVID versus ‘become low’ post=-COVID [undefined in paper] | Anxiety in persons without depression | OR | 0·94  (0·55-1·63) | NS |  |
|  |  | Television use ‘consistently low’ pre COVID versus ‘become high’ post-COVID [undefined in paper] | Anxiety in persons without depression | OR | 1·73  (0·48-2·02) | <0·05 |  |
|  |  | Television use ‘consistently low’ pre COVID versus ‘consistently high’ post COVID [undefined in paper] | Anxiety in persons without depression | OR | 1·13  (0·87-1·48) | NS |  |
|  |  | Television use ‘consistently low’ pre-COVID versus ‘become low’ post=-COVID [undefined in paper] | Loneliness in persons with depression | OR | 1·76  (0·60-5·18) | NS |  |
|  |  | Television use ‘consistently low’ pre COVID versus ‘become high’ post-COVID [undefined in paper] | Loneliness in persons with depression | OR | 1·37  (0·96-1·96) | NS |  |
|  |  | Television use ‘consistently low’ pre COVID versus ‘consistently high’ post COVID [undefined in paper] | Loneliness in persons with depression | OR | 0·84  (0·53-1·33) | NS |  |
|  |  | Television use ‘consistently low’ pre-COVID versus ‘become low’ post=-COVID [undefined in paper] | Sadness in persons with depression | OR | 1·28  (0·40-4·10) | NS |  |
|  |  | Television use ‘consistently low’ pre COVID versus ‘become high’ post-COVID [undefined in paper] | Sadness in persons with depression | OR | 1·61  (1·12-2·23) | <0·05 |  |
|  |  | Television use ‘consistently low’ pre COVID versus ‘consistently high’ post COVID [undefined in paper] | Sadness in persons with depression | OR | 0·87  (0·54-1·41) | NS |  |
|  |  | Television use ‘consistently low’ pre-COVID versus ‘become low’ post=-COVID [undefined in paper] | Anxiety in persons with depression | OR | 1·03  (0·31-3·40) | NS |  |
|  |  | Television use ‘consistently low’ pre COVID versus ‘become high’ post-COVID [undefined in paper] | Anxiety in persons with depression | OR | 1·58  (1·12-2·23) | <0·05 |  |
|  |  | Television use ‘consistently low’ pre COVID versus ‘consistently high’ post COVID [undefined in paper] | Anxiety in persons with depression | OR | 0·79  (0·46-1·36) | NS |  |
|  | Bird et al. | Screen time | Overall mental health | Unstandardised regression coefficient | 0·21  (-0·13-0·55) | NS | Age, sitting time |
|  | Hodes et al. | Objectively measured smartphone screen time | Depression | Pearson’s r | 0·14 | <0·05 | NA |
|  |  |  | Trait anxiety | Pearson’s r | 0·01 | NS | NA |
|  |  |  | Mobile phone attachment | Pearson’s r | 0·32 | <0·001 | NA |
|  | Conroy et al. | Screen time before bed | Mood change | Unstandardised regression coefficient | 0·23  (-0·04-0·48) | NS | Age, sex, frontline worker status, working from home |
|  | Lawrence et al. | Screen time | Depression | Unstandardised regression coefficient | 0·74  (NR) | NS | Demographic variables |
|  |  | Screen time | Anxiety | Unstandardised regression coefficient | 0·93  (NR) | 0·04 |  |
|  | Zhou et al. | Screen time (>1.43hrs a day with <1.43hrs/day as the reference group) | Depression | OR | 1·54  (1·03-2·30) | 0.035 | Gender, grade, profession, place of hometown, only child or not, monthly family income, parents’ marital status, smoking and drinking |
|  | Robbins et al. | TV use (less TV with same as the reference group) | Anxiety about COVID-19 | Prevalence ratio | 0·8  (0·5-1·1) | NS | Gender and health conditions that were noted to have a significant univariate association with the outcome |
|  |  | TV use (more TV with same as the reference group) | Anxiety about COVID-19 | Prevalence ratio | 1·4  (1·2-1·6) | <0.001 |  |
|  |  | TV use (less TV with same as the reference group) | Depression about COVID-19 | Prevalence ratio | 0·9  (0·6-1·4) | NS |  |
|  |  | TV use (more TV with same as the reference group) | Depression about COVID-19 | Prevalence ratio | 1·3  (1·1-1·5) | 0.001 |  |
|  | Zhang et al. | Screen time (>4hr/day with <2hr/day as the reference) | Depressive symptoms | OR | 0·54  (0·43-0·65) | <0.01 | ‘Cofounding variables' (not explicitly stated) |
|  | Fraser et al. | TV use | Concern for one's future | Unstandardised regression coefficient | -0·14  (SD=0·14) | NS | NR |
|  |  | Social media use | Concern for one's future | Unstandardised regression coefficient | 0·3  (SD=0·14) | NS |  |
|  |  | Gaming | Concern for one's future | Unstandardised regression coefficient | -0·17  (SD=1·) | NS |  |
|  |  | TV use | Concern for society | Unstandardised regression coefficient | 0·3  (SD=0·15) | 0.04 |  |
|  |  | Social media use | Concern for society | Unstandardised regression coefficient | -0·25  (0·14) | NS |  |
|  |  | Gaming | Concern for society | Unstandardised regression coefficient | -0·12  (SD=0·13) | NS |  |
|  | Zarco-Alpuente et al. | TV | Lockdown impact on mental health | Unstandardised regression coefficient | 0·16 | <0·001 | NR |
|  |  | TV | PANAS - positive effect | Unstandardised regression coefficient | -0·09 | <0·05 |  |
|  |  | TV | PANAS - negative effect | Unstandardised regression coefficient | NR | NS |  |
|  |  | Online sexual activities | Lockdown impact on mental health | Unstandardised regression coefficient | 0·13 | <0·05 |  |
|  |  | Online sexual activities | PANAS - positive effect | Unstandardised regression coefficient | NR | NS |  |
|  |  | Online sexual activities | PANAS - negative effect | Unstandardised regression coefficient | NR | NS |  |
|  |  | Video games | Lockdown impact on mental health | Unstandardised regression coefficient | NR | NS |  |
|  |  | Video games | PANAS - positive effect | Unstandardised regression coefficient | NR | NS |  |
|  |  | Video games | PANAS - negative effect | Unstandardised regression coefficient | NR | NS |  |
|  |  | Social networks | Lockdown impact on mental health | Unstandardised regression coefficient | NR | NS |  |
|  |  | Social networks | PANAS - positive effect | Unstandardised regression coefficient | NR | NS |  |
|  |  | Social networks | PANAS - negative effect | Unstandardised regression coefficient | -0·08 | <0·05 |  |
|  |  | Online shopping | Lockdown impact on mental health | Unstandardised regression coefficient | 0·16 | <0·01 |  |
|  |  | Online shopping | PANAS - positive effect | Unstandardised regression coefficient | NR | NS |  |
|  |  | Online shopping | PANAS - negative effect | Unstandardised regression coefficient | 0·15 | <0·05 |  |
|  |  | Instant messaging | Lockdown impact on mental health | Unstandardised regression coefficient | NR | NS |  |
|  |  | Instant messaging | PANAS - positive effect | Unstandardised regression coefficient | -0·1 | <0·01 |  |
|  |  | Instant messaging | PANAS - negative effect | Unstandardised regression coefficient | NR | NS |  |
|  | Le et al. | Screen time | Mood | Spearman r | 0·06 | NS | NA |
|  |  | Screen time | Anxiety | Spearman r | 0·17 | NS |  |
|  |  | Screen time | Worry | Spearman r | 0·12 | NS |  |
|  | Mon-Lopez et al. | Screen time | Emotional state | Pearson's r | -0·23 | <0·01 | NA |
|  | Yang et al. | Social media use | Post-traumatic stress | β of direct effect | 0·52  (0·35-0·76) | < 0·001 | Significant socio-demographic variables of SMA symptoms (age, current marital status, and educational level) |
|  |  | Social media use | Boredom | β of direct effect | 0· 46  (0.33-0·77) | < 0·001 |  |
|  |  | Social media use | Emotional loneliness | β of direct effect | 0·52  (0·36-0·77) | < 0·001 |  |
|  |  | Social media use | Social loneliness | β of direct effect | 0·54  (0·38-0·80) | < 0·001 |  |
|  |  | Social media use | Post-traumatic stress | β of indirect effect | 0·01  (-0·01-0·30) | NS |  |
|  |  | Social media use | Boredom | β of indirect effect | 0·03  (0·0.1-0·06) | < 0·05 |  |
|  |  | Social media use | Emotional loneliness | β of indirect effect | 0·01  (-0·01-0·02) | NS |  |
|  |  | Social media use | Social loneliness | β of indirect effect | -0·01  (-0·02=-0·001) | < 0·05 |  |
|  |  | Internet game use | Post-traumatic stress | β of direct effect | 0·43  (0·31-0·60) | < 0·001 |  |
|  |  | Internet game use | Boredom | β of direct effect | 0.39  (0·27-0·56) | < 0·001 |  |
|  |  | Internet game use | Emotional loneliness | β of direct effect | 0·44  (0·32-0·61) | < 0·001 |  |
|  |  | Internet game use | Social loneliness | β of direct effect | 0·43  (0·31-0·60) | < 0·001 |  |
|  |  | Internet game use | Post-traumatic stress | β of indirect effect | 0·001  (-0·01-0·01) | NS |  |
|  |  | Internet game use | Boredom | β of indirect effect | 0·04  (0·003-0·09) | < 0·05 |  |
|  |  | Internet game use | Emotional loneliness | β of indirect effect | -0·02  (-0·05-0·01) | NS |  |
|  |  | Internet game use | Social loneliness | β of indirect effect | -0·001  (-·03-0·1) | NS |  |
| Sleep/fatigue | Koohsari et al. | TV viewing time during workday | Total fatigue | Unstandardised regression coefficient | 0·16  (-0·44-0·76) | NS | Age, sex, marital status, highest education, gross annual household income, and baseline fatigue |
|  |  | PC use sitting time during workday | Subjective fatigue | Unstandardised regression coefficient | 0·05  (-0·25-0·35) | NS |  |
|  |  | PC use sitting time during workday | Total fatigue | Unstandardised regression coefficient | -0·06  (-0·61-0·48) | NS |  |
|  |  | TV viewing time | Subjective fatigue | Unstandardised regression coefficient | 0·11  (-0·19-0·40) | NS |  |
|  |  | TV viewing time | Motivation | Unstandardised regression coefficient | 0·03  (-0·11-0·17) | NS |  |
|  |  | TV viewing time | Total fatigue | Unstandardised regression coefficient | 0·33  (-0·20-0·85) | NS |  |
|  |  | PC use sitting time | Subjective fatigue | Unstandardised regression coefficient | -0·20  (-0·31-0·27) | NS |  |
|  |  | PC use sitting time | Motivation | Unstandardised regression coefficient | 0·03  (-0·11-0·17) | NS |  |
|  |  | PC use sitting time | Total fatigue | Unstandardised regression coefficient | -0·05  (-0·57-0·46) | NS |  |
|  | Le et al. | Screen time | Hours of sleep | Spearman’s r | 0.2 | NS | NA |
| General health | Suka et al. | Television use | General health status | OR | 0·99  (0·90-1·09) | NS | Gender, age, occupation, marital status, income, and general health status |
|  |  | Digital media exposure | General health status | OR | 1·14  (1·03-1·27) | <0·05 |  |
| Physical activity | Koohsari et al. | PC use sitting time during workday | Physical activity | Unstandardized regression coefficient | 0·03  (-0·09-0·14) | NS | Age, sex, marital status, highest education, gross annual household income, and baseline fatigue |
|  |  | TV viewing time | Physical activity | Unstandardized regression coefficient | 0·07  (-0·04-0·18) | NS |  |
|  |  | PC use sitting time | Physical activity | Unstandardized regression coefficient | 0·04  (-0·07-0·15) | NS |  |
|  | Abdulsalam et al. | Screen time | Physical activity | Kruksal-Wallis | NR | 0.004 | NA |
|  | Helbach and Stahlmann | Screen time | Physical activity | Unstandardised regression coefficient | -0.08  (-0.13—0.03) | <0.001 | Sex |
|  |  | Smartphone use | Physical activity | Unstandardised regression coefficient | -0.10  (-0.21-0.002) | NS | Sex, age, second job |
|  |  | TV use | Physical activity | Unstandardised regression coefficient | -0.15  (-0.26—0.04) | 0.01 | Sex, vocational degree |
|  |  | PC/computer/tablet | Physical activity | Unstandardised regression coefficient | -0.05  (-0.14-0.038) | NS | Sex; free time; second job; working hours |
|  |  | Gaming | Physical activity | Unstandardised regression coefficient | -0.21  (-0.37—0.04) | 0.01 | Sex |
|  |  | Social media | Physical activity | Unstandardised regression coefficient | -0.06  (-0.12—0.003) | 0.04 | Sex;age |
| Weight gain/BMI | Agurto et al. | Sitting or lying in front of a screen | Gain weight vs lose weight vs stayed saame | Chi squared | NR | 0·002 | NA |
|  | Saxena et al. | Screen time | BMI | T test | 0·13 | NS | NA |

**Supplementary Table 3: Reported associations between screen time and any outcome in children**

| **Type of association** | **Author(s)** | **Type of screen time**  **(dependent variable)** | **Type of correlation**  **(independent variable)** | **Effect size type** | **Effect size**  **(95% CI)** | **p-value** | **Adjustments** |
| --- | --- | --- | --- | --- | --- | --- | --- |
| Diet | Hashem et al. | Mobile extra screen time | Decrease or loss of appetite | Spearman's correlation | 0·012 | NS | NA |
|  |  | Mobile extra screen time | Increase of appetite | Spearman's correlation | 0·125 | <0·001 | NA |
|  |  | Mobile extra screen time | Increase sweets and unhealthy food | Spearman's correlation | 0·074 | 0·04 | NA |
|  |  | Mobile extra screen time | Does not care about eating vegetables and fruits | Spearman's correlation | 0·093 | 0·01 | NA |
|  |  | Mobile extra screen time | Decrease in his regular protein intake | Spearman's correlation | 0·106 | 0·003 | NA |
|  |  | Mobile extra screen time | Frequent snacks between meals | Spearman's correlation | 0·059 | NS | NA |
|  |  | Mobile extra screen time | Late snacks during night | Spearman's correlation | 0·158 | <0·001 | NA |
|  |  | TV extra screen time | Decrease or loss of appetite | Spearman's correlation | 0·072 | <0·05 | NA |
|  |  | TV extra screen time | Increase of appetite | Spearman's correlation | 0·043 | NS | NA |
|  |  | TV extra screen time | Increase sweets and unhealthy food | Spearman's correlation | 0·114 | 0·002 | NA |
|  |  | TV extra screen time | Does not care about eating vegetables and fruits | Spearman's correlation | 0·024 | NS | NA |
|  |  | TV extra screen time | Decrease in his regular protein intake | Spearman's correlation | 0·047 | NS | NA |
|  |  | TV extra screen time | Frequent snacks between meals | Spearman's correlation | 0·076 | 0·04 | NA |
|  |  | TV extra screen time | Late snacks during night | Spearman's correlation | 0·09 | 0·01 | NA |
|  |  | Laptop extra screen time | Decrease or loss of appetite | Spearman's correlation | -0·139 | <0·001 | NA |
|  |  | Laptop extra screen time | Increase of appetite | Spearman's correlation | 0·162 | <0·001 | NA |
|  |  | Laptop extra screen time | Increase sweets and unhealthy food | Spearman's correlation | 0·022 | NS | NA |
|  |  | Laptop extra screen time | Does not care about eating vegetables and fruits | Spearman's correlation | 0·102 | 0·005 | NA |
|  |  | Laptop extra screen time | Decrease in his regular protein intake | Spearman's correlation | 0·015 | NS | NA |
|  |  | Laptop extra screen time | Frequent snacks between meals | Spearman's correlation | 0·198 | <0·001 | NA |
|  |  | Laptop extra screen time | Late snacks during night | Spearman's correlation | 0·069 | NS | NA |
|  |  | Video game extra screen time | Decrease or loss of appetite | Spearman's correlation | -0·023 | NS | NA |
|  |  | Video game extra screen time | Increase of appetite | Spearman's correlation | 0·123 | <0·001 | NA |
|  |  | Video game extra screen time | Increase sweets and unhealthy food | Spearman's correlation | 0·047 | NS | NA |
|  |  | Video game extra screen time | Does not care about eating vegetables and fruits | Spearman's correlation | 0·045 | NS | NA |
|  |  | Video game extra screen time | Decrease in his regular protein intake | Spearman's correlation | 0·031 | NS | NA |
|  |  | Video game extra screen time | Frequent snacks between meals | Spearman's correlation | 0·065 | NS | NA |
|  |  | Video game extra screen time | Late snacks during night | Spearman's correlation | 0·087 | 0·016 | NA |
|  |  | Remote learning | Decrease or loss of appetite | Spearman's correlation | 0·112 | 0·002 | NA |
|  |  | Remote learning | Increase of appetite | Spearman's correlation | -0·065 | NS | NA |
|  |  | Remote learning | Increase sweets and unhealthy food | Spearman's correlation | 0·001 | NS | NA |
|  |  | Remote learning | Does not care about eating vegetables and fruits | Spearman's correlation | 0·097 | 0·007 | NA |
|  |  | Remote learning | Decrease in his regular protein intake | Spearman's correlation | 0·075 | 0·038 | NA |
|  |  | Remote learning | Frequent snacks between meals | Spearman's correlation | -0·083 | 0·022 | NA |
|  |  | Remote learning | Late snacks during the night | Spearman's correlation | 0·041 | NS | NA |
| Eye | Liu et al. a | Overall screen time | Myopic symptoms vs no myopic symptoms | OR | 1·26  (1·21-1·31) | <0·001 | Sex, school grade, rural location. |
|  |  | Computer vs TV | Myopic symptoms vs no myopic symptoms | OR | 1·81  (1·05-3·12) | 0·032 |  |
|  |  | Smartphone vs TV | Myopic symptoms vs no myopic symptoms | OR | 2·02  (1·19-3·43) | 0·009 |  |
|  |  | Multiple devices vs TV | Myopic symptoms vs no myopic symptoms | OR | 1·56  (0·90-2·68) | NS |  |
|  | Liu et al. b | e-learning screen use | e-learning screen use and progression of myopic symptoms | OR | 1·07  (1·06-1·09) | <0·001 | Sex, grade, and location |
|  | Mohan et al. | Digital deice usage | Digital eye strain | OR | 3.6  (1.7-7.6) | <0.001 | NR |
| Mental health | Szwarcwald et al. | Screen time | At least two problems from frequent sadness, frequent irritability, and sleep problems | OR | 2·51  (NR) | <0·001 | Sex, age group, skin color, family financial difficulties, and food insecurity |
|  | Alves et al. | Leisure screen time | State anxiety (Overweight or obese) | Pearson's r | 0·2 | <0·001 | Child age, sex, SES and GDM exposure |
|  |  | Leisure screen time | Positive affect  (Overweight or obese) | Pearson's r | 0·02 | NS |  |
|  |  | Leisure screen time | Negative affect  (Overweight or obese) | Pearson's r | 0·62 | NS |  |
|  |  | Leisure screen time | State anxiety  (health weight) | Pearson's r | 0·28 | <0·05 |  |
|  |  | Leisure screen time | Positive affect  (health weight) | Pearson's r | -0·44 | NS |  |
|  |  | Leisure screen time | Negative affect  (health weight) | Pearson's r | 0·38 | <0·05 |  |
|  | Farah et al. | Screen exposure | Child stress | Multiple mediation analysis | 0·179 | <0·05 | NR |
|  | Xiao et al. | Online study time | Mood disturbance | Unstandardised regression coefficient | 0·43 | <0·05 | Sex, grade(age), physical activity |
|  |  | Other screen time | Mood disturbance | Unstandardised regression coefficient | 1·6 | NS |  |
|  | Sikorska et al. | Playing online games | Depression | Pearson's r | 0.122 | <0.05 | NA |
|  |  | Playing online games | Anxiety | Pearson's r | 0.112 | <0.05 | NA |
|  |  | Playing online games | Stress | Pearson's r | 0.114 | <0.05 | NA |
|  |  | Playing online games | Resilience | Pearson's r | -0.01 | NS | NA |
|  |  | Playing online games | Emotional well-being | Pearson's r | -0.072 | NS | NA |
|  |  | Playing online games | Psychological well-being | Pearson's r | -0.06 | NS | NA |
|  |  | Playing online games | Social wellbeing | Pearson's r | -0.066 | NS | NA |
|  |  | Internet browsing | Depression | Pearson's r | 0.211 | <0.01 | NA |
|  |  | Internet browsing | Anxiety | Pearson's r | 0.205 | <0.01 | NA |
|  |  | Internet browsing | Stress | Pearson's r | 0.187 | <0.01 | NA |
|  |  | Internet browsing | Resilience | Pearson's r | 0.049 | NS | NA |
|  |  | Internet browsing | Emotional well-being | Pearson's r | -0.157 | <0.01 | NA |
|  |  | Internet browsing | Psychological well-being | Pearson's r | -0.126 | <0.05 | NA |
|  |  | Internet browsing | Social wellbeing | Pearson's r | -0.112 | <0.05 | NA |
|  |  | TV | Depression | Pearson's r | 0.164 | <0.01 | NA |
|  |  | TV | Anxiety | Pearson's r | 0.162 | <0.01 | NA |
|  |  | TV | Stress | Pearson's r | 0.202 | <0.01 | NA |
|  |  | TV | Resilience | Pearson's r | 0.064 | NS | NA |
|  |  | TV | Emotional well-being | Pearson's r | -0.075 | NS | NA |
|  |  | TV | Psychological well-being | Pearson's r | -0.073 | NS | NA |
|  |  | TV | Social wellbeing | Pearson's r | -0.071 | NS | NA |
|  |  | Social media | Depression | Pearson's r | 0.233 | <0.01 | NA |
|  |  | Social media | Anxiety | Pearson's r | 0.233 | <0.01 | NA |
|  |  | Social media | Stress | Pearson's r | 0.192 | <0.01 | NA |
|  |  | Social media | Resilience | Pearson's r | 0.029 | NS | NA |
|  |  | Social media | Emotional well-being | Pearson's r | -0.115 | <0.05 | NA |
|  |  | Social media | Psychological well-being | Pearson's r | -0.103 | <0.05 | NA |
|  |  | Social media | Social wellbeing | Pearson's r | -0.085 | NS | NA |
|  | Giannini et al. | Screen time | Calmness and screen time >2 -<5hrs (<2hrs is the reference) | OR | 1.9 (0.5-8.0) | NS | None |
|  |  | Screen time | Calmness and screen time >5 - <8hrs (<2hrs is the reference) | OR | 0.8  (0.1-3.7) | NS | None |
|  |  | Screen time | Calmness and screen time >8hrs (<2hrs is the reference) | OR | 1  (0.2-3.7) | NS | None |
|  |  | Screen time | Anxiety and screen time >2 -<5hrs (<2hrs is the reference) | OR | 0.7  (0.2-2.2) | NS | None |
|  |  | Screen time | Anxiety and screen time >5 - <8hrs (<2hrs is the reference) | OR | 1.4  (0.4-5.0) | NS | None |
|  |  | Screen time | Anxiety and screen time >8hrs (<2hrs is the reference) | OR | 1.7  (0.5-5.0) | NS | None |
|  |  | Screen time | Sadness and screen time >2 -<5hrs (<2hrs is the reference) | OR | 0.6  (0.2-1.9) | NS | None |
|  |  | Screen time | Sadness and screen time >5 - <8hrs (<2hrs is the reference) | OR | 0.4  (0.1-1.6) | NS | None |
|  |  | Screen time | Sadness and screen time >8hrs (<2hrs is the reference) | OR | 0.8  (0.3-2.5) | NS | None |
|  |  | Screen time | Fear and screen time >2 -<5hrs (<2hrs is the reference) | OR | 0.6  (0.2-1.2) | NS | None |
|  |  | Screen time | Fear and screen time >5 - <8hrs (<2hrs is the reference) | OR | 0.1  (0-0.5) | 0.01 | None |
|  |  | Screen time | Fear and screen time >8hrs (<2hrs is the reference) | OR | 0.3  (0.1-0.9) | 0.04 | None |
|  |  | Screen time | Depression and screen time >2 -<5hrs (<2hrs is the reference) | OR | 0.4  (0.1-1.6) | NS | None |
|  |  | Screen time | Depression and screen time >5 - <8hrs (<2hrs is the reference) | OR | 0.5  (0.2-1.6) | NS | None |
|  |  | Screen time | Depression and screen time >8hrs (<2hrs is the reference) | OR | 0.9  (0.3-2.9) | NS | None |
|  |  | Screen time | Anger and screen time >2 -<5hrs (<2hrs is the reference) | OR | 0.4  (0.1-1.3) | NS | None |
|  |  | Screen time | Anger and screen time >5 - <8hrs (<2hrs is the reference) | OR | 0.3  (0.1-1.1) | NS | None |
|  |  | Screen time | Anger and screen time >8hrs (<2hrs is the reference) | OR | 0.5  (0.2-1.6) | NS | None |
|  | Aguilar-Farias et al. | Screen time | Being more affectionate and total screen time | Unstandardised regression coefficient | 0.01  (-0.04-0.07) | NS | Child and caregiver's age; change in working conditions due to the pandemic; parental tiredness; parental struggling to concentrate; parental having difficulties related to work |
|  |  | Screen time | Being more restless and total screen time | Unstandardised regression coefficient | 0.06  (0-0.13) | NS |  |
|  |  | Screen time | Being more aggressive and total screen time | Unstandardised regression coefficient | 0.12  (0.04-0.19) | <0.01 |  |
|  |  | Screen time | Being more irritable and total screen time | Unstandardised regression coefficient | 0.12  (0.06-0.19) | <0.001 |  |
|  |  | Screen time | Having more temper tantrums and total screen time | Unstandardised regression coefficient | 0.1  (0.03-0.17) | <0.01 |  |
|  |  | Screen time | Being more frustrated and total screen time | Unstandardised regression coefficient | 0.13  (0.06-0.19) | <0.001 |  |
|  |  | Screen time | Being more worried and total screen time | Unstandardised regression coefficient | 0.05  (-0.01-0.12) | NS |  |
|  |  | Screen time | Being more sad and total screen time | Unstandardised regression coefficient | 0.06  (-0.01-0.13) | NS |  |
|  |  | Screen time | Being more sensitive and total screen time | Unstandardised regression coefficient | 0.08  (0.01-0.15) | <0.05 |  |
|  |  | Screen time | Being more afraid and total screen time | Unstandardised regression coefficient | 0.01  (-0.07-0.08) | NS |  |
|  | Kim et al. | TV | Subjective stress index and tv time | Pearson's r | -0.002 | NS | NA |
|  |  | TV | Behaviour problems index and tv time | Pearson's r | 0.06 | NS |  |
|  |  | Tablet time | Subjective stress index and tv time | Pearson's r | 0.17 | <0.05 |  |
|  |  | Tablet time | Behaviour problems index and tv time | Pearson's r | 0.22 | <0.05 |  |
|  |  | Smartphone time | Subjective stress index and tv time | Pearson's r | 0.13 | NS |  |
|  |  | Smartphone time | Behaviour problems index and tv time | Pearson's r | 0.17 | <0.05 |  |
|  | Stokes et al. | TV | COVID worries | OR | 1.2  (0.8-1.7) | NS | COVID-19 stress, child age, child sex, ADHD medication use, externalizing disorder, internalizing disorder, neighbourhood socio-economic status, and financial insecurity. |
|  |  | Social media | COVID worries | OR | 0.8  (0.5-1.2) | NS |  |
|  |  | Gaming | COVID worries | OR | 1.6  (1.1-2.3) | 0.02 |  |
|  |  | TV | COVID stress | OR | 1.4  (0.9-2.0) | NS |  |
|  |  | Social media | COVID stress | OR | 2.1  (1.3-3.3) | 0.003 |  |
|  |  | Gaming | COVID stress | OR | 0.9  (0.6-1.3) | NS |  |
|  | Chen et al. | Smartphone use | Psychological distress | Pearson's r | 0.2 | <0.01 | NA |
|  | Breidokiene et al. | Screen time for education | Child's emotional well-being | Bivariate correlation | -0.02 | NS | NA |
|  |  | Screen time for leisure | Child's emotional well-being | Bivariate correlation | -0.06 | NS | NA |
| Physical activity | Alves et al. | Leisure screen time | Sedentary time  (Overweight/obese) | Pearson's r | 0·71 | <0·05 | Age, sex, SES and GDM exposure |
|  |  | Leisure screen time | Moderate/vigorous physical activity  (Overweight/obese) | Pearson's r | -0·39 | NS |  |
|  |  | Leisure screen time | Sedentary time  (Healthy weight) | Pearson's r | 0·41 | <0·05 |  |
|  |  | Leisure screen time | Moderate/vigorous physical activity  (Healthy weight) | Pearson's r | -0·02 | NS |  |
|  | Cachon-Zagalaz et al. | Daily use of digital screens | Daily physical activity | Unstandardised regression coefficient | -0·118  (NR) | NS | Age, hours of sleep per day, daily activities |
|  | Jáuregui et al. | Overall screen time | Physical activity and total screen time | Unstandardised regression coefficient | -0.18  (-0.25; -0.11) | <0.05 | age; sex; sleep time; having someone to play with; access to electronic devices; screen in the bedroom; rules limiting screen time; caregiver chrematistics; availability of toys; availability of space to play; socioeconomic level; region |
|  | Breidokiene et al. | Screen time for education | Physical activity | Bivariate correlation | -0.16 | < 0.01 | NA |
|  |  | Screen time for education | Time outdoors | Bivariate correlation | -0.16 | < 0.01 | NA |
|  |  | Screen time for leisure | Physical activity | Bivariate correlation | -0.21 | < 0.01 | NA |
|  |  | Screen time for leisure | Time outdoors | Bivariate correlation | -0.22 | < 0.01 | NA |
| Parental health | Farah et al. | Screen exposure | Parental stress (COVID 19) | Chi square | -0·058 | NS | NA |
|  |  | Screen exposure | Parent employment status | Chi square | 0·005 | NS | NA |
|  |  | Screen exposure | Parent anxiety | Chi square | 0·099 | NS | NA |
|  |  | Screen exposure | Parental screen use | Chi square | 0·167 | <0·05 | NA |
|  | McCormack et al. | TV | >2hr/day and parental anxiety (low versus high) | OR | 1·47  (0·83-2·61) | NS | child gender, parent gender, child age, parent age, gross annual household income, parent employment status, parent education, parent ethnicity, marital status, number of children in household, and dog ownership |
|  |  | Video gaming | >2hr/day and parental anxiety (low versus high) | OR | 1·78  (1·02-3·11) | <0·05 |  |
|  |  | Using screen based devices | >2hr/day and parental anxiety (low versus high) | OR | 1·04  (0·62-1·73) | NS |  |
|  | Xiao et al. | Online study time | Online study time and conflicts with parents | Unstandardized regression coefficient | 0·02  (NR) | <0·05 | Sex, grade(age), physical activity |
|  |  | Other screen time | Other screen time and conflicts with parents | Unstandardized regression coefficient | 0·06  (NR) | <0·01 | NR |
|  | McArthur et al. | Screen time (child report) | Awareness of media use | Unstandardized regression coefficient | -0·43  (-1·47-0·61) | NS | NR |
|  |  | Screen time (child report) | Screen time rules | Unstandardized regression coefficient | -3·20  (-5·30;-2·19) | <0·05 | NR |
|  |  | Screen time (child report) | Pandemic impact on resources | Unstandardized regression coefficient | 2·06  (0·57-3·54) | <0·05 | NR |
|  |  | Screen time (child report) | Maternal stress | Unstandardized regression coefficient | 0·07  (-0·04-0·18) | NS | NR |
|  |  | Screen time (child report) | Difficulty balancing homelife | Unstandardized regression coefficient | -0·89  (-2·61-0·84) | NS | NR |
|  |  | Screen time (child report) | Job/income loss | Unstandardized regression coefficient | 0·43  (-1·06-1·92) | NS | NR |
|  |  | Screen time (child report) | Difficulty obtaining childcare | Unstandardized regression coefficient | -1·22  (-2·87-0·44) | NS | NR |
|  |  | Screen time (maternal report) | Awareness of media use | Unstandardized regression coefficient | -3·37  (-4·20; -2·54) | <0·05 | NR |
|  |  | Screen time (maternal report) | Screen time rules | Unstandardized regression coefficient | -3·81  (-5·43; -2·19) | <0·05 | NR |
|  |  | Screen time (maternal report) | Pandemic impact on resources | Unstandardized regression coefficient | 0·26  (-0·95-1·47) | NS | NR |
|  |  | Screen time (maternal report) | Maternal stress | Unstandardized regression coefficient | 0·21  (0·12-0·30) | <0·05 | NR |
|  |  | Screen time (maternal report) | Difficulty balancing homelife | Unstandardized regression coefficient | 1·13  (-0·26-2·52) | NS | NR |
|  |  | Screen time (maternal report) | Job/income loss | Unstandardized regression coefficient | -1·10  (-2·29-0·10) | NS | NR |
|  |  | Screen time (maternal report) | Difficulty obtaining childcare | Unstandardized regression coefficient | 0·27  (-1·08-1·62) | NS | NR |
|  | Breidokiene et al. | Screen time for education | Parental distress | Bivariate correlation | 0.03 | NR | NA |
|  |  | Screen time for leisure | Parental distress | Bivariate correlation | 0.01 | NR | NA |
| Physiology | Nassar et al. | Screen time | Change in BMI | T-test | 0·23 | NS | NR |
|  | Hadianfard et al. | Screen time | Being overweight and obese (2nd screen time quartile with the 1st quartile as the reference) | OR | 1.8  (0.53-6.04) | NS | Age, sex, grade, family size, access to the yard |
|  |  | Screen time | Being overweight and obese (3nd screen time quartile with the 1st quartile as the reference) | OR | 2.6  (0.43-15.9) | NS |  |
|  |  | Screen time | Being overweight and obese (4th screen time quartile with the 1st quartile as the reference) | OR | 1.59  (0.14-17.32) | NS |  |
| Sleep | Lim et al. | Non-academic screen time | Sleep duration | Pearson's r | -0·41 | <0·01 | NR |
|  | Cachon-Zagalaz et al. | Daily use of digital screens | Hours of sleep per day | Unstandardized regression coefficient | -0·40 | <0·01 | Age, daily PA, daily activities |
|  | Windiani et al. | Screen time | Sleep disorders | OR | 3·80  (1·09-13·1) | 0·02 | Crude |
|  | Jáuregui et al. | Screen time | Sleep time | Unstandardized regression coefficient | 6.35  (1.68-11.03) | <0.05 | NR |
|  | Kim et al. | TV | Sleep problems | Pearson's r | -0.005 | NS | NA |
|  |  | Tablet time | Sleep problems | Pearson's r | 0.172 | <0.05 |  |
|  |  | Smartphone time | Sleep problems | Pearson's r | 0.298 | <0.001 |  |
| Problematic screen time behaviours | Donati et al. | Gaming time | Gaming disorder symptoms | Pearson's r | 0.43 | <0.001 | NA |
|  | Chen et al. | Smartphone use | Problematic smartphone use | Pearson's r | 0.35 | <0.01 | NA |
|  |  | Smartphone use | Problematic social media use | Pearson's r | 0.29 | <0.01 | NA |
|  |  | Smartphone use | Problematic gaming | Pearson's r | 0.25 | <0.01 | NA |
